# Supplementary figures and images for: Healthy lifestyle, endoscopic screening, and colorectal cancer incidence and mortality in the United States: A nationwide cohort study
Source: PLoS Med. 2021 Feb 1;18(2):e1003522. doi: 10.1371/journal.pmed.1003522 (PMC7886195; doi:10.1371/journal.pmed.1003522)

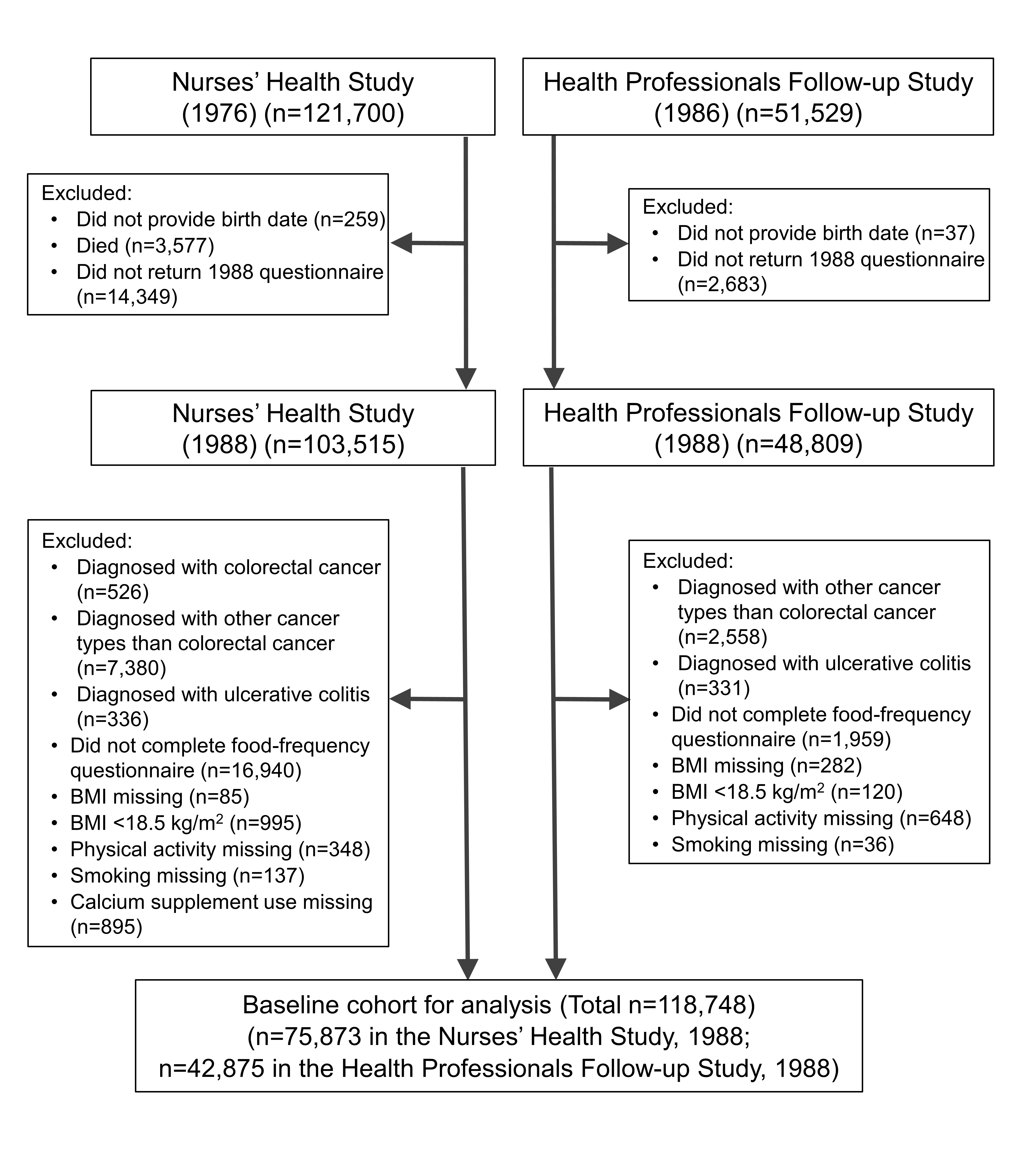

Supplement: S1 Fig — (TIF) [file pmed.1003522.s002.tif]

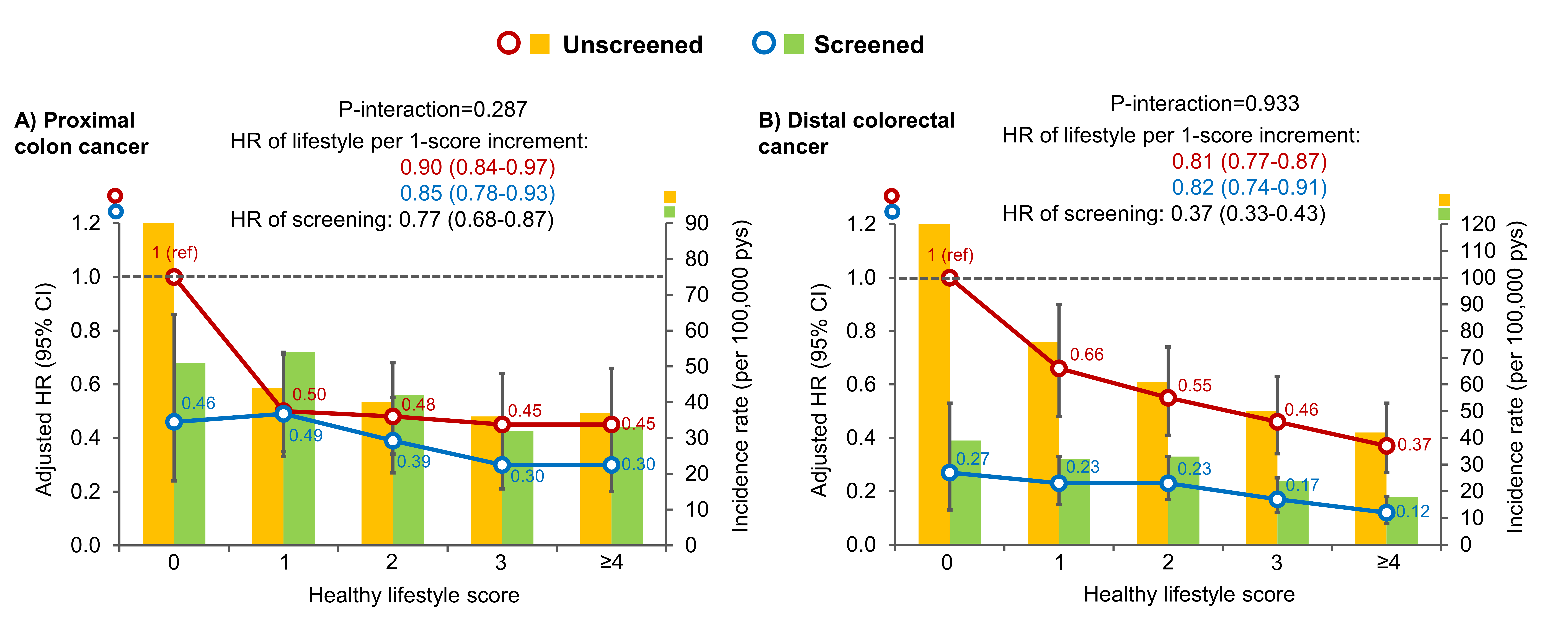

Supplement: S2 Fig — Association of healthy lifestyle score with incidence of proximal colon cancer (panel A) and distal colorectal cancer (panel B) according to endoscopic screening status and the corresponding age-adjusted incidence rates. Healthy lifestyle score (range, 0–5) was defined as the number of the 5 healthy lifestyle factors: normal body weight (BMI, ≥18.5 and <25.0 kg/m2), never smoking or past smoking with pack-years <5, moderate-to-vigorous intensity activity for ≥30 minutes per day, none-to-moderate alcohol intake (<1 drink [14 g alcohol]/d for women and <2 drinks/d for men), and meeting at least 3 of the 6 dietary recommendations by the WCRF/AICR Third Expert Report 2018, which included red meat <0.5 serving/d, processed meat <0.2 serving/d, dietary fiber ≥30 g/d, dairy products ≥3 servings/d, whole grains ≥48 g/d or account for at least half of total grains, and calcium supplement use. Multivariable Cox proportional hazards regression was used to calculate the HRs and 95% CIs while adjusting for age, calendar period, sex, ethnicity, current multivitamins use, regular aspirin use, family history of CRC, and menopausal status and hormone use (women only). P-heterogeneity = 0.175 among unscreened participants and 0.349 among screened participants. BMI, body mass index; CI, confidence interval; CRC, colorectal cancer; HR, hazard ratio; pys, person-years; WCRF/AICR, World Cancer Research Fund/American Institute for Cancer Research. (TIF) [file pmed.1003522.s003.tif]

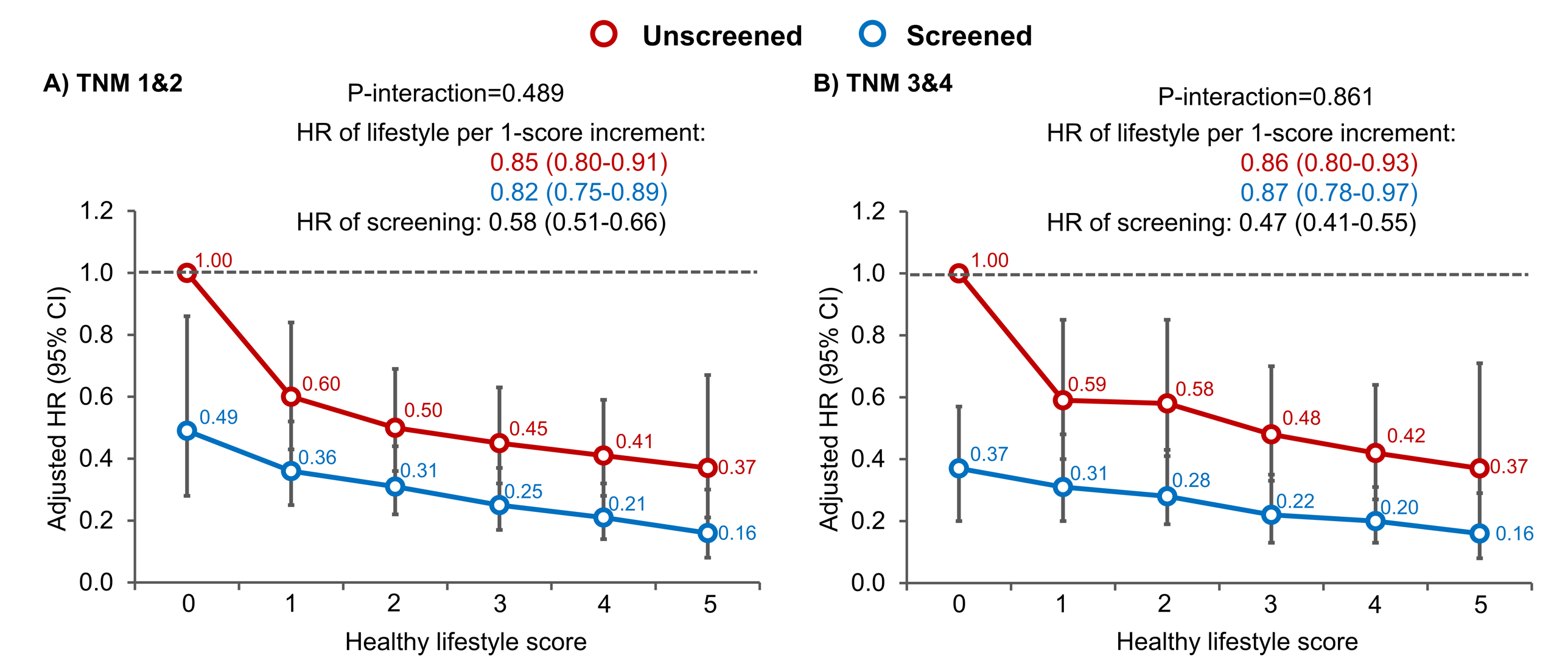

Supplement: S3 Fig — Association of healthy lifestyle score with incidence of CRC by TNM stage (panel A: TNM 1 and 2; panel B: TNM 3 and 4) according to endoscopic screening status. Healthy lifestyle score (range, 0–5) was defined as the number of the 5 healthy lifestyle factors: normal body weight (BMI, ≥18.5 and <25.0 kg/m2), never smoking or past smoking with pack-years <5, moderate-to-vigorous intensity activity for ≥30 minutes per day, none-to-moderate alcohol intake (<1 drink [14 g alcohol]/d for women and <2 drinks/d for men), and meeting at least 3 of the 6 dietary recommendations by the WCRF/AICR Third Expert Report 2018, which included red meat <0.5 serving/d, processed meat <0.2 serving/d, dietary fiber ≥30 g/d, dairy products ≥3 servings/d, whole grains ≥48 g/d or account for at least half of total grains, and calcium supplement use. Multivariable Cox proportional hazards regression was used to calculate the HRs and 95% CIs while adjusting for age, calendar period, sex, ethnicity, current multivitamins use, regular aspirin use, family history of CRC, and menopausal status and hormone use (women only). BMI, body mass index; CI, confidence interval; CRC, colorectal cancer; HR, hazard ratio; TNM, tumor–node–metastasis; WCRF/AICR, World Cancer Research Fund/American Institute for Cancer Research. (TIF) [file pmed.1003522.s004.tif]

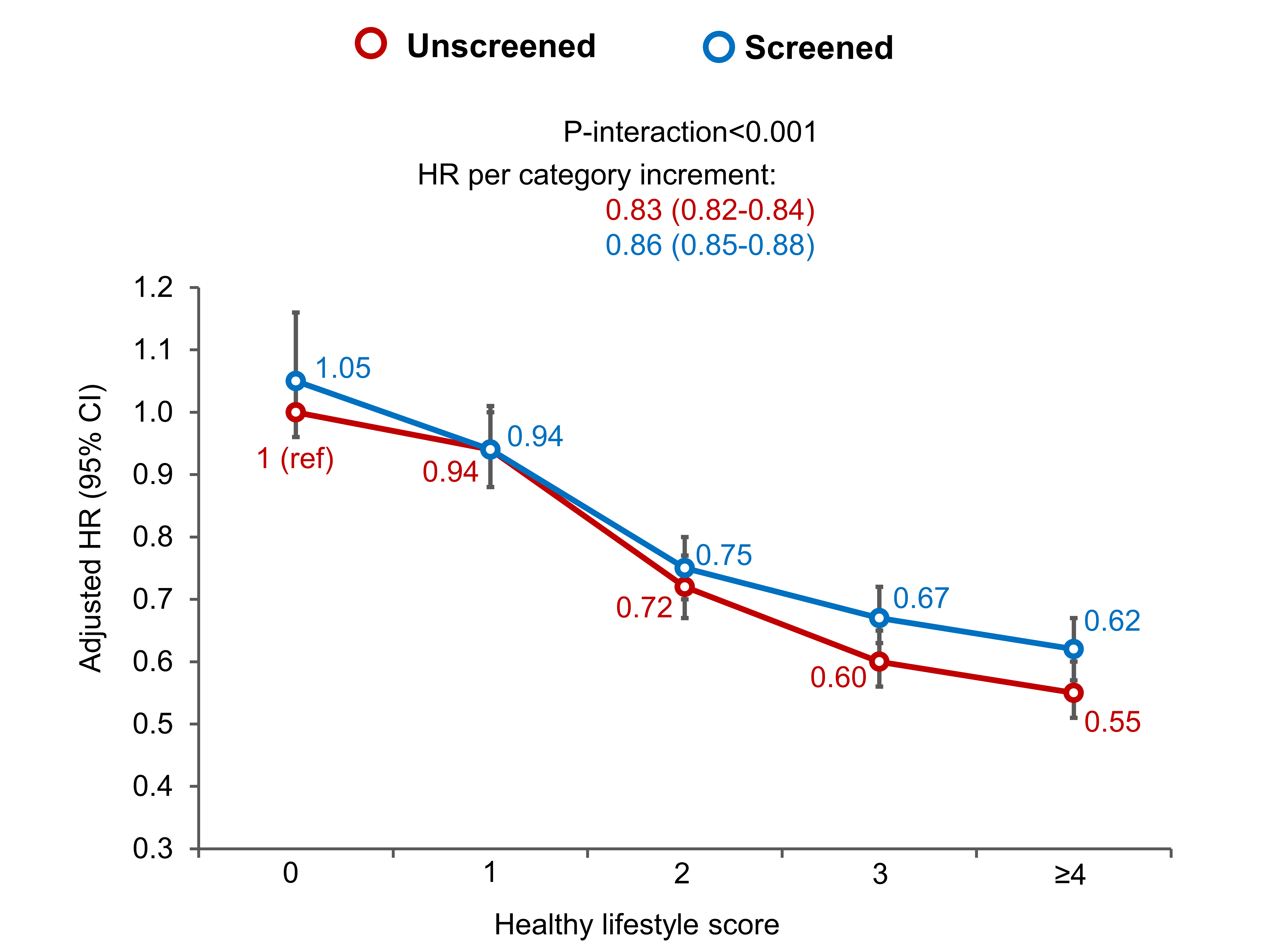

Supplement: S4 Fig — Healthy lifestyle score (range, 0–5) was defined as the number of the 5 healthy lifestyle factors: normal body weight (BMI, ≥18.5 and <25.0 kg/m2), never smoking or past smoking with pack-years <5, moderate-to-vigorous intensity activity for ≥30 minutes per day, none-to-moderate alcohol intake (<1 drink [14 g alcohol]/d for women and <2 drinks/d for men), and meeting at least 3 of the 6 dietary recommendations by the WCRF/AICR Third Expert Report 2018, which included red meat <0.5 serving/d, processed meat <0.2 serving/d, dietary fiber ≥30 g/d, dairy products ≥3 servings/d, whole grains ≥48 g/d or account for at least half of total grains, and calcium supplement use. Multivariable Cox proportional hazards regression was used to calculate the HRs and 95% CIs while adjusting for age, calendar period, sex, ethnicity, current multivitamins use, regular aspirin use, family history of CRC, menopausal status and hormone use (women only), diagnoses of cardiovascular disease and type 2 diabetes, physical exam for disease screening, mammography for breast cancer screening (women only), and prostate-specific antigen testing for prostate cancer screening (men only). BMI, body mass index; CI, confidence interval; CRC, colorectal cancer; HR, hazard ratio; WCRF/AICR, World Cancer Research Fund/American Institute for Cancer Research. (TIF) [file pmed.1003522.s005.tif]

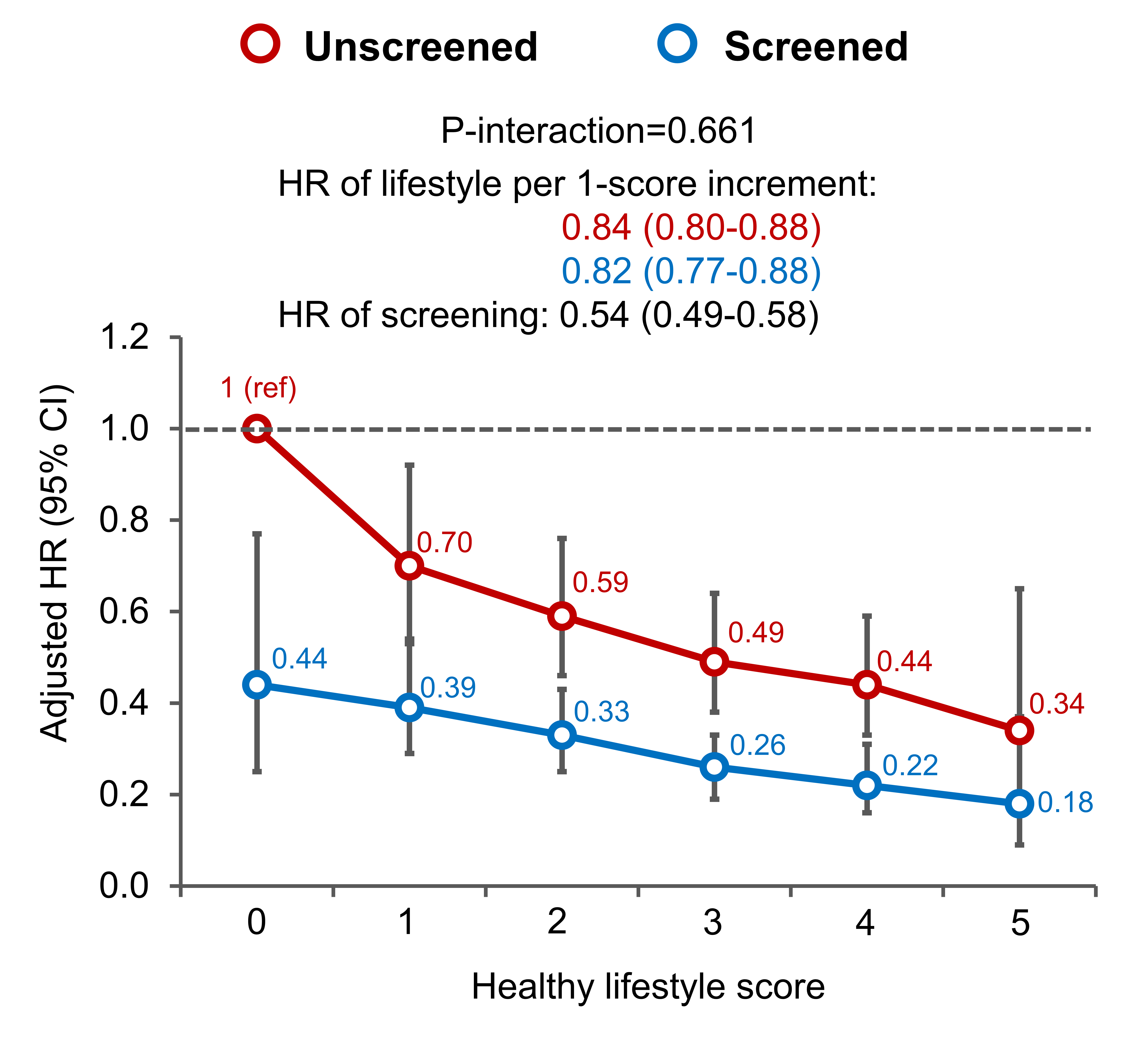

Supplement: S5 Fig — The five 5-categorical lifestyle factors included BMI (18.5–24.9, 25.0–27.4, 27.5–29.5, 30.0–34.9, and ≥35.0 kg/m2; scored 5–1, respectively), smoking (never, past smoking with pack-years <5, past smoking with pack-years ≥5, current smoker with pack-years <20, and current smoker with pack-years ≥20; scored 5–1, respectively), physical activity (moderate-to-vigorous intensity activity for 0, 0.1–0.9, 1.0–3.4, 3.5–5.9, and ≥6 hours per week; scored 1–5, respectively), alcohol intake (0, 0.1–13.9, 14–20.9, 21–27.9, and ≥28 g/d; scored 5–1, respectively), and number of the 6 healthy dietary components recommendations by the WCRF/AICR Third Expert Report 2018 (0–1, 2, 3, 4, and 5–6; scored 1–5, respectively). The 6 healthy dietary components included red meat <0.5 serving/d, processed meat <0.2 serving/d, dietary fiber ≥30 g/d, dairy products ≥3 servings/d, whole grains ≥48 g/d or account for at least half of total grains, and calcium supplement use. Sum of the 5 scores (range, 5–25) was then categorical into 5 levels (5–10, 11–13, 14–16, 17–19, 20–22, and 23–25). These 5 levels were defined as the new healthy lifestyle score (0, 1, 2, 3, 4, and 5). Multivariable Cox proportional hazards regression was used to calculate the HRs and 95% CIs while adjusting for age, calendar period, sex, ethnicity, current multivitamins use, regular aspirin use, family history of CRC, and menopausal status and hormone use (women only). Error bars indicate 95% CIs. BMI, body mass index; CI, confidence interval; CRC, colorectal cancer; HR, hazard ratio; WCRF/AICR, World Cancer Research Fund/American Institute for Cancer Research. (TIF) [file pmed.1003522.s006.tif]
