# Supplementary material for: Healthy lifestyle, endoscopic screening, and colorectal cancer incidence and mortality in the United States: A nationwide cohort study
Source: PLoS Med. 2021 Feb 1;18(2):e1003522. doi: 10.1371/journal.pmed.1003522 (PMC7886195; doi:10.1371/journal.pmed.1003522)
Supplement: S1 Table — (DOCX) [file pmed.1003522.s007.docx]

**S1 Table. Associations of individual lifestyle factors with colorectal cancer incidence and mortality according to endoscopic screening status**

| Lifestyle factors | HR (95% CI)^*^ | | | |
| --- | --- | --- | --- | --- |
|  | Colorectal cancer incidence | | Colorectal cancer mortality | |
|  | Unscreened | Screened | Unscreened | Screened |
| Body mass index, kg/m^2^ |  |  |  |  |
| 18.5-24.9 | 1.88 (1.66-2.14) | 1.00 (reference) | 2.36 (1.81-3.07) | 1.00 (reference) |
| 25.0-27.4 | 2.19 (1.91-2.51) | 1.08 (0.92-1.27) | 2.36 (1.81-3.08) | 1.23 (0.92-1.65) |
| 27.5-29.5 | 2.25 (1.92-2.62) | 1.19 (0.98-1.44) | 2.61 (1.96-3.46) | 1.17 (0.83-1.63) |
| 30.0-34.9 | 2.26 (1.91-2.68) | 1.29 (1.03-1.61) | 2.27 (1.69-3.04) | 1.12 (0.79-1.59) |
| ≥35.0 | 2.42 (1.91-3.07) | 1.61 (1.12-2.31) | 2.51 (1.77-3.54) | 1.83 (1.21-2.76) |
| Per category | 1.07 (1.04-1.17) | 1.10 (1.04-1.17) | 1.01 (0.95-1.07) | 1.09 (1.00-1.19) |
| P-interaction | 0.387 | | 0.123 | |
| <25 vs. ≥25 | 0.85 (0.77-0.93) | 0.86 (0.75-0.98) | 0.98 (0.83-1.16) | 0.81 (0.63-1.04) |
|  | 0.85 (0.79-0.92) | | 0.92 (0.80-1.06) | |
| Smoking, pack-years |  |  |  |  |
| Never | 1.88 (1.64-2.15) | 1.00 (reference) | 1.85 (1.47-2.31) | 1.00 (reference) |
| Past smoker, <5 | 1.93 (1.62-2.30) | 1.04 (0.83-1.28) | 1.92 (1.44-2.57) | 0.80 (0.55-1.17) |
| Past smoker, ≥5 | 2.41 (2.11-2.75) | 1.26 (1.09-1.46) | 2.61 (2.10-3.24) | 1.24 (0.97-1.57) |
| Current smoker, <20 | 1.71 (1.13-2.60) | 0.82 (0.34-1.98) | 1.34 (0.59-3.06) | 0.84 (0.21-3.40) |
| Current smoker, ≥20 | 2.00 (1.65-2.43) | 1.09 (0.77-1.55) | 2.56 (1.87-3.50) | 1.21 (0.68-2.14) |
| Per category | 1.05 (1.01-1.09) | 1.08 (1.01-1.14) | 1.11 (1.05-1.18) | 1.09 (0.98-1.20) |
| P-interaction | 0.914 | | 0.461 | |
| Never or past smoker with pack-years <5 vs. past smoker with pack-years ≥5 or current smoker | 0.82 (0.75-0.90) | 0.82 (0.71-0.93) | 0.73 (0.62-0.85) | 0.77 (0.62-0.96) |
|  | 0.82 (0.76-0.88) | | 0.74 (0.65-0.84) | |
| Alcohol intake, g/d |  |  |  |  |
| 0 | 1.53 (1.26-1.85) | 1.00 (reference) | 1.73 (1.28-2.35) | 1.00 (reference) |
| 0.1-13.9 | 1.84 (1.55-2.19) | 0.94 (0.79-1.13) | 1.87 (1.42-2.47) | 0.87 (0.65-1.17) |
| 14-20.9 | 2.03 (1.62-2.55) | 0.97 (0.74-1.26) | 1.83 (1.25-2.68) | 1.21 (0.81-1.81) |
| 21-27.9 | 2.27 (1.74-2.94) | 1.08 (0.78-1.49) | 2.57 (1.70-3.89) | 0.92 (0.54-1.59) |
| ≥28 | 2.59 (2.08-3.23) | 1.34 (1.03-1.74) | 1.91 (1.31-2.81) | 0.79 (0.48-1.30) |
| Per category | 1.14 (1.09-1.18) | 1.08 (1.02-1.15) | 1.05 (0.97-1.12) | 0.98 (0.88-1.09) |
| P-interaction | 0.169 | | 0.313 | |
| None-to-moderate vs. heavy | 0.76 (0.67-0.86) | 0.72 (0.60-0.86) | 0.98 (0.78-1.23) | 0.88 (0.65-1.21) |
|  | 0.75 (0.68-0.83) | | 0.95 (0.79-1.14) | |
| Physical activity, h/week^†^ |  |  |  |  |
| 0 | 1.00 (reference) | 0.60 (0.41-0.87) | 1.00 (reference) | 0.61 (0.34-1.09) |
| 0.1-0.9 | 0.83 (0.67-1.03) | 0.43 (0.34-0.54) | 0.81 (0.58-1.13) | 0.36 (0.25-0.53) |
| 1.0-3.4 | 0.76 (0.62-0.94) | 0.42 (0.34-0.53) | 0.72 (0.51-1.01) | 0.39 (0.28-0.56) |
| 3.5-5.9 | 0.75 (0.59-0.95) | 0.44 (0.34-0.57) | 0.82 (0.56-1.19) | 0.36 (0.23-0.55) |
| ≥6 | 0.85 (0.67-1.09) | 0.35 (0.26-0.46) | 0.65 (0.43-0.99) | 0.30 (0.19-0.48) |
| Per category | 0.97 (0.92-1.01) | 0.94 (0.88-1.00) | 0.94 (0.86-1.01) | 0.92 (0.83-1.03) |
| P-interaction | 0.319 | | 0.798 | |
| ≥3.5 vs. <3.5 | 0.98 (0.88-1.10) | 0.92 (0.79-1.07) | 0.96 (0.79-1.17) | 0.86 (0.66-1.10) |
|  | 0.96 (0.87-1.05) | | 0.92 (0.79-1.08) | |
| NO. of healthy dietary components^‡^ |  |  |  |  |
| 0-1 | 1.00 (reference) | 0.56 (0.50-0.64) | 1.00 (reference) | 0.52 (0.42-0.64) |
| 2 | 0.99 (0.90-1.10) | 0.46 (0.40-0.53) | 1.06 (0.89-1.27) | 0.52 (0.41-0.65) |
| 3 | 0.84 (0.73-0.97) | 0.51 (0.44-0.60) | 0.99 (0.79-1.25) | 0.46 (0.34-0.61) |
| 4 | 1.03 (0.84-1.27) | 0.35 (0.26-0.47) | 1.31 (0.95-1.81) | 0.38 (0.23-0.61) |
| 5-6 | 0.59 (0.34-1.03) | 0.68 (0.45-1.03) | 0.15 (0.02-1.03) | 0.78 (0.40-1.53) |
| Per category | 0.95 (0.91-1.00) | 0.94 (0.88-0.99) | 1.01 (0.93-1.09) | 0.96 (0.86-1.07) |
| P-interaction | 0.587 | | 0.511 | |
| ≥3 vs. <3 | 0.88 (0.78-0.98) | 0.93 (0.80-1.08) | 1.00 (0.83-1.21) | 0.88 (0.68-1.13) |
|  | 0.90 (0.82-0.98) | | 0.96 (0.82-1.11) | |

Abbreviations: HR, hazard ratio; CI, confidence interval.

^*^HRs and 95% CIs were calculated while adjusting for the other 4 of the 5 lifestyle factors, age, calendar period, sex, ethnicity, current multivitamins use, regular aspirin use, family history of colorectal cancer, menopausal status and hormone use (women only) in incidence analysis and additionally diagnoses of cardiovascular disease and type 2 diabetes in mortality analysis.

^†^Physical activity was of moderate-to-vigorous intensity requiring the expenditure of ≥3 metabolic equivalents per hour.

^‡^Healthy dietary components included red meat <0.5 serving/d, processed meat <0.2 serving/d, dietary fiber ≥30 g/d, dairy products ≥3 servings/d, whole grains ≥48 g/d or account for at least half of total grains, and calcium supplement use, as recommended by the World Cancer Research Fund/American Institute for Cancer Research Third Expert Report 2018.
