# Supplementary material for: Healthy lifestyle, endoscopic screening, and colorectal cancer incidence and mortality in the United States: A nationwide cohort study
Source: PLoS Med. 2021 Feb 1;18(2):e1003522. doi: 10.1371/journal.pmed.1003522 (PMC7886195; doi:10.1371/journal.pmed.1003522)
Supplement: S2 Table — (DOCX) [file pmed.1003522.s008.docx]

**S2 Table. Associations of individual lifestyle factors with incidence of proximal colon cancer and distal colorectal cancer according to endoscopic screening status**

| Lifestyle factors | HR (95% CI)^*^ | | | |
| --- | --- | --- | --- | --- |
|  | Proximal colon cancer | | Distal colorectal cancer | |
|  | Unscreened | Screened | Unscreened | Screened |
| Body mass index, kg/m^2^ |  |  |  |  |
| 18.5-24.9 | 1.35 (1.12-1.62) | 1.00 (reference) | 2.85 (2.32-3.49) | 1.00 (reference) |
| 25.0-27.4 | 1.38 (1.12-1.71) | 0.97 (0.77-1.22) | 3.43 (2.76-4.26) | 1.20 (0.91-1.57) |
| 27.5-29.5 | 1.37 (1.06-1.75) | 1.11 (0.84-1.47) | 3.41 (2.68-4.35) | 1.44 (1.05-1.98) |
| 30.0-34.9 | 1.50 (1.15-1.96) | 1.22 (0.89-1.66) | 3.22 (2.47-4.20) | 1.43 (0.98-2.08) |
| ≥35.0 | 1.30 (0.85-1.97) | 1.68 (1.04-2.71) | 4.13 (2.93-5.81) | 1.49 (0.75-2.94) |
| Per category | 1.02 (0.95-1.08) | 1.09 (1.01-1.18) | 1.07 (1.01-1.13) | 1.14 (1.03-1.25) |
| P-interaction | 0.172 | | 0.278 | |
| <25 vs. ≥25 | 0.97 (0.83-1.13) | 0.92 (0.76-1.11) | 0.83 (0.73-0.95) | 0.76 (0.61-0.96) |
|  | 0.95 (0.84-1.07) | | 0.81 (0.73-0.91) | |
| Smoking, pack-years |  |  |  |  |
| Never | 1.29 (1.05-1.58) | 1.00 (reference) | 2.92 (2.34-3.63) | 1.00 (reference) |
| Past smoker, <5 | 1.60 (1.22-2.10) | 1.14 (0.84-1.54) | 2.34 (1.76-3.10) | 1.16 (0.82-1.65) |
| Past smoker, ≥5 | 1.67 (1.37-2.04) | 1.27 (1.03-1.56) | 3.53 (2.84-4.38) | 1.30 (1.01-1.66) |
| Current smoker, <20 | 1.09 (0.51-2.34) | 0.36 (0.05-2.57) | 2.95 (1.71-5.10) | 1.81 (0.66-4.91) |
| Current smoker, ≥20 | 1.35 (0.99-1.85) | 1.27 (0.80-2.01) | 3.04 (2.27-4.06) | 0.92 (0.48-1.76) |
| Per category | 1.05 (0.98-1.11) | 1.09 (1.01-1.18) | 1.04 (0.99-1.10) | 1.08 (0.97-1.19) |
| P-interaction | 0.783 | | 0.876 | |
| Never or past smoker with pack-years <5 vs. past smoker with pack-years ≥5 or current smoker | 0.85 (0.73-0.99) | 0.82 (0.68-0.99) | 0.81 (0.71-0.92) | 0.82 (0.65-1.02) |
|  | 0.84 (0.74-0.95) | | 0.81 (0.73-0.91) | |
| Alcohol intake, g/d |  |  |  |  |
| 0 | 1.10 (0.82-1.46) | 1.00 (reference) | 2.16 (1.58-2.94) | 1.00 (reference) |
| 0.1-13.9 | 1.38 (1.07-1.77) | 0.99 (0.76-1.27) | 2.53 (1.90-3.37) | 0.93 (0.68-1.26) |
| 14-20.9 | 1.06 (0.71-1.57) | 1.10 (0.75-1.60) | 3.26 (2.31-4.60) | 0.96 (0.61-1.50) |
| 21-27.9 | 1.73 (1.14-2.60) | 1.03 (0.64-1.67) | 2.88 (1.93-4.31) | 1.00 (0.58-1.74) |
| ≥28 | 1.86 (1.32-2.63) | 1.43 (0.98-2.09) | 3.65 (2.60-5.13) | 1.34 (0.87-2.07) |
| Per category | 1.12 (1.04-1.20) | 1.09 (1.00-1.19) | 1.14 (1.07-1.20) | 1.08 (0.97-1.19) |
| P-interaction | 0.653 | | 0.359 | |
| None-to-moderate vs. heavy | 0.85 (0.69-1.05) | 0.68 (0.54-0.87) | 0.70 (0.59-0.83) | 0.72 (0.54-0.97) |
|  | 0.78 (0.66-0.91) | | 0.71 (0.61-0.82) | |
| Physical activity, h/week^†^ |  |  |  |  |
| 0 | 1.00 (reference) | 0.86 (0.47-1.57) | 1.00 (reference) | 0.50 (0.28-0.89) |
| 0.1-0.9 | 0.82 (0.56-1.20) | 0.63 (0.42-0.94) | 0.92 (0.68-1.23) | 0.32 (0.23-0.46) |
| 1.0-3.4 | 0.73 (0.49-1.07) | 0.60 (0.41-0.90) | 0.91 (0.67-1.22) | 0.33 (0.24-0.46) |
| 3.5-5.9 | 0.86 (0.57-1.31) | 0.66 (0.43-1.01) | 0.79 (0.56-1.10) | 0.34 (0.23-0.50) |
| ≥6 | 0.85 (0.54-1.32) | 0.43 (0.26-0.69) | 0.93 (0.67-1.31) | 0.32 (0.21-0.48) |
| Per category | 0.99 (0.91-1.07) | 0.91 (0.82-1.00) | 0.97 (0.91-1.04) | 0.97 (0.87-1.08) |
| P-interaction | 0.156 | | 0.841 | |
| ≥3.5 vs. <3.5 | 1.09 (0.91-1.32) | 0.89 (0.72-1.11) | 0.92 (0.79-1.08) | 0.97 (0.76-1.24) |
|  | 1.00 (0.87-1.16) | | 0.94 (0.82-1.07) | |
| NO. of healthy dietary components^‡^ |  |  |  |  |
| 0-1 | 1.00 (reference) | 0.86 (0.71-1.03) | 1.00 (reference) | 0.40 (0.33-0.49) |
| 2 | 1.10 (0.92-1.31) | 0.71 (0.58-0.88) | 0.94 (0.81-1.09) | 0.30 (0.23-0.38) |
| 3 | 1.03 (0.83-1.28) | 0.78 (0.61-0.99) | 0.74 (0.60-0.90) | 0.34 (0.25-0.44) |
| 4 | 0.80 (0.54-1.18) | 0.51 (0.33-0.78) | 1.10 (0.83-1.44) | 0.24 (0.14-0.39) |
| 5-6 | 0.53 (0.20-1.44) | 1.06 (0.59-1.91) | 0.59 (0.28-1.25) | 0.28 (0.11-0.67) |
| Per category | 0.97 (0.89-1.05) | 0.93 (0.85-1.02) | 0.93 (0.87-1.00) | 0.88 (0.78-0.98) |
| P-interaction | 0.598 | | 0.313 | |
| ≥3 vs. <3 | 0.92 (0.76-1.11) | 0.92 (0.75-1.13) | 0.83 (0.71-0.98) | 0.86 (0.66-1.10) |
|  | 0.92 (0.80-1.06) | | 0.84 (0.73-0.96) | |

Abbreviations: HR, hazard ratio; CI, confidence interval.

^*^HRs and 95% CIs were calculated while adjusting for other 4 of the 5 lifestyle factors, age, calendar period, sex, ethnicity, current multivitamins use, regular aspirin use, family history of colorectal cancer, menopausal status and hormone use (women only).

^†^Physical activity was of moderate-to-vigorous intensity requiring the expenditure of ≥3 metabolic equivalents per hour.

^‡^Healthy dietary components included red meat <0.5 serving/d, processed meat <0.2 serving/d, dietary fiber ≥30 g/d, dairy products ≥3 servings/d, whole grains ≥48 g/d or account for at least half of total grains, and calcium supplement use, as recommended by the World Cancer Research Fund/American Institute for Cancer Research Third Expert Report 2018.
