# Supplementary material for: Healthy lifestyle, endoscopic screening, and colorectal cancer incidence and mortality in the United States: A nationwide cohort study
Source: PLoS Med. 2021 Feb 1;18(2):e1003522. doi: 10.1371/journal.pmed.1003522 (PMC7886195; doi:10.1371/journal.pmed.1003522)
Supplement: S5 Table — (DOCX) [file pmed.1003522.s011.docx]

**S5 Table. Population-attributable risk estimates for colorectal cancer incidence and mortality with colonoscopic screening and healthy lifestyle^*^ separately and in combination**

| Exposures | % PAR (95% CI)^†^ | |
| --- | --- | --- |
|  | Colorectal cancer incidence | Colorectal cancer mortality |
| Colonoscopic screening | 38 (34-41) | 55 (49-60) |
| Healthy lifestyle score ≥2 | 7 (5-9) | 4 (0-8) |
| Healthy lifestyle score ≥3 | 17 (12-21) | 19 (10-28) |
| Healthy lifestyle score ≥4 | 26 (18-34) | 20 (2-37) |
| Healthy lifestyle score =5 | 30 (11-48) | - |
| Colonoscopic screening & healthy lifestyle score ≥2 | 43 (37-47) | 57 (48-64) |
| Colonoscopic screening & healthy lifestyle score ≥3 | 48 (41-54) | 63 (53-72) |
| Colonoscopic screening & healthy lifestyle score ≥4 | 54 (46-61) | 64 (51-74) |
| Colonoscopic screening & healthy lifestyle score =5 | 58 (43-68) | - |

Abbreviations: PAR, population-attributable risk; CI, confidence interval.

^*^Healthy lifestyle score (range, 0-5) was defined as the number of the 5 healthy lifestyle factors: normal body weight (body mass index, ≥18.5 and <25.0 kg/m^2^), never smoking or past smoking with pack-years <5, moderate-to-vigorous intensity activity for ≥30 minutes per day, none-to-moderate alcohol intake (<1 drink [14g alcohol]/d for women and <2 drinks/d for men), and meeting at least 3 of the 6 dietary recommendations by the World Cancer Research Fund/American Institute for Cancer Research Third Expert Report 2018, which included red meat <0.5 serving/d, processed meat <0.2 serving/d, dietary fiber ≥30 g/d, dairy products ≥3 servings/d, whole grains ≥48 g/d or account for at least half of total grains, and calcium supplement use.

^†^PARs and 95% CIs were calculated while adjusting for age, calendar period, sex, ethnicity, current multivitamins use, regular aspirin use, family history of colorectal cancer, menopausal status and hormone use (women only) in incidence analysis and additionally diagnoses of cardiovascular disease and type 2 diabetes in mortality analysis.
