# Supplementary material for: Healthy lifestyle, endoscopic screening, and colorectal cancer incidence and mortality in the United States: A nationwide cohort study
Source: PLoS Med. 2021 Feb 1;18(2):e1003522. doi: 10.1371/journal.pmed.1003522 (PMC7886195; doi:10.1371/journal.pmed.1003522)
Supplement: S8 Table — (DOCX) [file pmed.1003522.s014.docx]

**S8 Table. Population-attributable risk estimates for colorectal cancer incidence with endoscopic screening and healthy lifestyle separately and in combination when healthy lifestyle score was defined by the 5 5-catgorocal lifestyle factors^*^**

| Exposures | % PAR (95% CI)^†^ |
| --- | --- |
| Endoscopic screening | 32 (31-33) |
| Healthy lifestyle score ≥2 | 6 (4-7) |
| Healthy lifestyle score ≥3 | 15 (11-19) |
| Healthy lifestyle score ≥4 | 23 (14-31) |
| Healthy lifestyle score =5 | 38 (9-62) |
| Endoscopic screening & healthy lifestyle score ≥2 | 36 (33-39) |
| Endoscopic screening & healthy lifestyle score ≥3 | 42 (37-47) |
| Endoscopic screening & healthy lifestyle score ≥4 | 48 (41-55) |
| Endoscopic screening & healthy lifestyle score =5 | 60 (38-74) |

Abbreviations: PAR, population-attributable risk; CI, confidence interval.

^*^The 5 5-categorical lifestyle factors included body mass index (18.5-24.9, 25.0-27.4, 27.5-29.5, 30.0-34.9, and ≥35.0 kg/m^2^; scored 5-1, respectively), smoking (never, past smoking with pack-years <5, past smoking with pack-years ≥5, current smoker with pack-years <20, and current smoker with pack-years ≥20; scored 5-1, respectively), physical activity (moderate-to-vigorous intensity activity for 0, 0.1-0.9, 1.0-3.4, 3.5-5.9, and ≥6 hours per week; scored 1-5, respectively), alcohol intake (0, 0.1-13.9, 14-20.9, 21-27.9, and ≥28 g/d; scored 5-1, respectively), and number of the 6 healthy dietary components recommendations by the World Cancer Research Fund/American Institute for Cancer Research Third Expert Report 2018 (0-1, 2, 3, 4, and 5-6; scored 1-5, respectively). The 6 healthy dietary components included red meat <0.5 serving/d, processed meat <0.2 serving/d, dietary fiber ≥30 g/d, dairy products ≥3 servings/d, whole grains ≥48 g/d or account for at least half of total grains, and calcium supplement use. Sum of the 5 scores (rang, 5-25) was then categorical into 5 levels (5-10, 11-13, 14-16, 17-19, 20-22, and 23-25). These 5 levels were defined as the new healthy lifestyle score (0, 1, 2, 3, 4, and 5).

^†^PARs and 95% CIs were calculated while adjusting for age, calendar period, sex, ethnicity, current multivitamins use, regular aspirin use, family history of colorectal cancer, menopausal status and hormone use (women only).
