# Supplementary material for: Healthy lifestyle, endoscopic screening, and colorectal cancer incidence and mortality in the United States: A nationwide cohort study
Source: PLoS Med. 2021 Feb 1;18(2):e1003522. doi: 10.1371/journal.pmed.1003522 (PMC7886195; doi:10.1371/journal.pmed.1003522)
Supplement: S1 Text — (DOCX) [file pmed.1003522.s015.docx]

**Title:** Healthy lifestyle, endoscopic screening, and colorectal cancer incidence and mortality

**Proposed authors:**

Kai Wang, Wenjie Ma, Kana Wu, Shuji Ogino, Andrew T. Chan, Edward Giovannucci, Mingyang Song

**Background:**

Colorectal cancer (CRC) is the third most commonly diagnosed cancer and the third leading cause of cancer death in the United States, accounting for 8.1% of new cancer cases and 8.3% of cancer deaths in 2018 [[1](#_ENREF_1)]. Endoscopic screening has been shown to reduce CRC incidence and mortality by identifying and removing precancerous polys and early cancers [[2-4](#_ENREF_2)]. According to the National Health Interview Survey, the percentage of recommendation-consistent CRC screening among U.S. adults aged 50 years and older has increased from 34% in 2000 to 63% in 2015, among which 60% reported having a sigmoidoscopy in the past 5 years or colonoscopy in the past 10 years [[5](#_ENREF_5)]. On the other hand, healthy diet and lifestyle practices, including no smoking, maintaining a healthy body weight, being physically active, and limiting alcohol drinking have also been shown to be associated with a substantial reduction in the incidence and mortality of CRC [[6-9](#_ENREF_6)]. According to reports in our cohorts, 71% and 37% of the CRC cases among men and women respectively could be potentially prevented by healthy lifestyle [[9](#_ENREF_9), [10](#_ENREF_10)], highlighting the essential role of lifestyle modification in the prevention of CRC. Although the associations of lifestyle factors and endoscopic screening with CRC risk have been compared and a stronger association was suggested for lifestyle [[8](#_ENREF_8)], it remains unknown whether maintenance of a healthy lifestyle after endoscopic screening could confer any additional benefit on CRC risk and mortality reduction. Also, the influence of lifestyle on death from other causes than CRC remains to be determined among individuals with and without endoscopic screening. Addressing these questions is of critical importance in the context of the ever-increasing use of endoscopic screening and the related concern about a “health certificate effect” after CRC screening [[11](#_ENREF_11)].

**Objectives:**

1. To characterize the associations of healthy lifestyle practices with CRC incidence, CRC mortality, and mortality from other causes among individuals with and without endoscopic screening, separately. While endoscopic screening may substantially reduce CRC risk and mortality, given the limitations of screening, we expect to observe additional benefit of healthy lifestyle after endoscopic screening on CRC prevention and overall health. We thus hypothesize a protective association of healthy lifestyle with CRC incidence and mortality among individuals with endoscopic screening, though the association is expected to be weaker compared to that among those without endoscopic screening. We hypothesize a similar association of healthy lifestyle with mortality from other causes than CRC among individuals with and without endoscopic screening.
2. To examine the absolute amount and proportion of CRC cases and deaths, among individuals with and without endoscopic screening separately, attributable to lifestyle risk factors, including smoking, unhealthy body weight, physical inactivity, high alcohol drinking, and unhealthy diet.

**Study population:** NHS (1988-2014) and HPFS (1988-2014)

**Exclusion criteria:** History of CRC diagnosis at baseline; missing data on lifestyle factors and endoscopic screening at baseline; history of cancer, CVD, or diabetes (for mortality analysis only).

**Exposures:** We will include 5 lifestyle factors: BMI, cigarette smoking, alcohol consumption, physical activity, and diet. For each factor, we will define a binary criterion, by which the participants received a score of 1 if they met the criterion and 0 otherwise. Specifically, the criteria include a BMI of ≥18.5 and <25.0 kg/m^2^, never smoking, physical activity for ≥30 minutes per day of moderate-to-vigorous intensity activity, and none-to-moderate alcohol intake (less than 1 drink [14 g alcohol] per day for women and less than 2 drinks per day for men). Diet will be assessed by consumption of the 6 dietary components recommended by the World Cancer Research Fund/American Institute for Cancer Research (WCRF/AICR) [[12](#_ENREF_12)], they are processed meat (<0.5 serving/d), red meat (<0.2 serving/d), whole grain (≥48 g/d), dietary fiber (≥30 g/d), dairy products (≥3 serving/d), and calcium supplement use (yes). If an individual has 3 or more of the 6 dietary components meat the recommended consumption criteria, he/she will be assigned to have healthy diet. An overall healthy lifestyle score (range, 0-5) was then calculated by summing the 5 scores, with a higher score indicating a healthier lifestyle.

**Outcome**: CRC incidence and mortality, and mortality due to other causes than CRC.

**Effect modifier:** History of a lower endoscopic screening, including colonoscopy and sigmoidoscopy (yes/no).

**Covariates:** age, ethnicity, current aspirin use, family history of CRC; current multivitamin use, menopausal status and hormone use (for women).

**Statistical analysis:**

1. We will estimate the relative risks (RR) for CRC incidence, CRC mortality, and mortality due to other causes than CRC associated with individual and combined lifestyle factors, among those with and without endoscopic screening separately, using Cox proportional hazards regression models.
2. We will calculate PARs of CRC incidence and mortality due to lifestyle risk factors, among individuals with and without endoscopic screening separately, using the prevalence of the lowest-risk lifestyle group (score 5) in the cohorts.
3. In secondary analyses, we will stratify by gender and age; examine the potential heterogeneity by tumor subsite (overall colon, proximal colon, distal colon, and rectum); and conduct a 4-8-year latency analysis to minimize the influence of reverse causation for mortality analysis.

**References:**

1. Siegel, R.L., K.D. Miller, and A. Jemal, *Cancer statistics, 2018.* CA Cancer J Clin, 2018. **68**(1): p. 7-30.

2. Selby, J.V., et al., *A case-control study of screening sigmoidoscopy and mortality from colorectal cancer.* N Engl J Med, 1992. **326**(10): p. 653-7.

3. Atkin, W.S., et al., *Once-only flexible sigmoidoscopy screening in prevention of colorectal cancer: a multicentre randomised controlled trial.* Lancet, 2010. **375**(9726): p. 1624-33.

4. Garcia-Albeniz, X., et al., *Effectiveness of Screening Colonoscopy to Prevent Colorectal Cancer Among Medicare Beneficiaries Aged 70 to 79 Years: A Prospective Observational Study.* Ann Intern Med, 2017. **166**(1): p. 18-26.

5. National Center for Health, S., *Health, United States*, in *Health, United States, 2015: With Special Feature on Racial and Ethnic Health Disparities*. 2016, National Center for Health Statistics (US): Hyattsville (MD).

6. Huxley, R.R., et al., *The impact of dietary and lifestyle risk factors on risk of colorectal cancer: a quantitative overview of the epidemiological evidence.* Int J Cancer, 2009. **125**(1): p. 171-80.

7. Kirkegaard, H., et al., *Association of adherence to lifestyle recommendations and risk of colorectal cancer: a prospective Danish cohort study.* Bmj, 2010. **341**: p. c5504.

8. Wei, E.K., et al., *A Comprehensive Model of Colorectal Cancer by Risk Factor Status and Subsite Using Data From the Nurses' Health Study.* Am J Epidemiol, 2017. **185**(3): p. 224-237.

9. Platz, E.A., et al., *Proportion of colon cancer risk that might be preventable in a cohort of middle-aged US men.* Cancer Causes Control, 2000. **11**(7): p. 579-88.

10. Erdrich, J., et al., *Proportion of colon cancer attributable to lifestyle in a cohort of US women.* Cancer Causes Control, 2015. **26**(9): p. 1271-1279.

11. Berstad, P., et al., *Long-term lifestyle changes after colorectal cancer screening: randomised controlled trial.* Gut, 2015. **64**(8): p. 1268-76.

12. World Cancer Research Fund/American Institute for Cancer, R., *Diet, nutrition, physical activity and cancer: a global perspective.* Continuous Update Project Expert Report, 2018.
