# Supplementary material for: Healthy lifestyle, endoscopic screening, and colorectal cancer incidence and mortality in the United States: A nationwide cohort study
Source: PLoS Med. 2021 Feb 1;18(2):e1003522. doi: 10.1371/journal.pmed.1003522 (PMC7886195; doi:10.1371/journal.pmed.1003522)
Supplement: S2 Text — (DOCX) [file pmed.1003522.s016.docx]

**S2 Text. Detailed Assessment and Statistical Analysis**

**Assessment of lifestyle and covariates**

Height was assessed at baseline. Body weight and cigarette smoking were assessed every 2 years. We also asked participants to recall their body weight at age 18 years in the NHS and 21 years in the HPFS. BMI was calculated as weight in kilograms divided by the square of height in meter (kg/m^2^). We assessed physical activity biennially by a validated questionnaire [1] and calculated the total hours per week for moderate-to-vigorous intensity activity (including brisk walking) that requires the expenditure of at least 3 metabolic equivalents (METs) per hour. A MET is the ratio of work metabolic rate to a standard resting metabolic rate of 1.0 (4.184 kJ) kg^−1^ hr^−1^ [2]. One MET is the resting metabolic rate while sitting [2]; activities between 3 and 6 METs are considered moderate intensity, while activities >6 METs are considered vigorous intensity [3]. Diet was assessed every 4 years with a validated food frequency questionnaire [4, 5] by inquiring the usual frequency of consumption for various foods during the previous year. Nutrient intake was calculated by multiplying the nutrient content of each food by the frequency of intake and then summing across all food items. Nutrient intakes were adjusted for total energy intake using the residual method [6]. Diet quality was assessed with the 6 major dietary recommendations in the World Cancer Research Fund/American Institute for Cancer Research (WCRF/AICR) Third Expert Report released in 2018 [7], which included a consumption of red meat <0.5 serving/d, processed meat <0.2 serving/d, dietary fiber ≥30 g/d, dairy products ≥3 servings/d, whole grains ≥48 g/d or accounting for at least half of total grains, and calcium supplement use. The number of the dietary recommendations met by a participant was assessed as a diet score (range, 0-6).

To capture long-term exposures and reduce random within-person variation, we calculated cumulative average levels of BMI, physical activity, diet score, and alcohol intake from our repeated questionnaires. For example, in the NHS, physical activity data collected on the 1988 questionnaire was used to assess risk of CRC that occurred between 1988 and 1990, the average of the 1988 and 1990 physical activity measurements was used for CRC risk between 1990 and 1992, the average of the 1988, 1990, and 1992 measurements was used for CRC risk between 1992 and 1994, and so on. Cigarette smoking was assessed by both pack-years and current status reported biennially. In mortality analysis, to minimize reverse causality resulting from weight loss caused by preexisting diseases, we used the maximum BMI reported throughout the follow-up [8]. For example, in the NHS, we used the maximum value of BMI reported for age 18 years and between the cohort enrollment in 1976 and 1988 to associate CRC mortality between 1988 and 1990, and the maximum BMI for age 18 and from 1976 to 1990 for CRC mortality between 1990 and 1992, and so on.

We also collected detailed information on potential confounders, including age, ethnicity, current multivitamins use, regular aspirin use, family history of CRC, menopausal status and hormone use (women only), diagnoses of cardiovascular disease (CVD) and type 2 diabetes, physical exam for disease screening, mammography for breast cancer screening (women only), and prostate-specific antigen testing for prostate cancer screening (men only). Regular aspirin use was defined as consumption of two or more aspirin tables per week for the NHS, and consumption of aspirin at least two times per week for the HPFS.

**Statistical analysis**

In this study, we only examined the endoscopies that were performed for screening purpose (including those performed for family history of CRC), whereas counted the endoscopies for investigation of symptoms (e.g., abdominal pain, diarrhea, or constipation), or for follow-up of a positive fecal occult blood test or abnormal imaging study in the non-endoscopic screening group. For the incidence analysis, as in previous studies [9, 10], we did not consider endoscopies that occurred within the same questionnaire cycle as CRC diagnosis as an endoscopy exposure, since such examinations were likely performed for the diagnosis and management of CRC. We thus stopped updating endoscopy status on the biennial questionnaire before the diagnosis of CRC, death from any cause, or end of follow-up, whichever came first. For the mortality analysis, endoscopy status was updated up to and including the time of CRC diagnosis, prior to death from any cause, or the last follow-up cycle, whichever came first.

We evaluated whether the associations between healthy lifestyle and CRC differed by endoscopic screening status by including a cross-product term between healthy lifestyle score (continuous) and endoscopic screening status in the model. In the multivariable analyses, we adjusted for several potential confounding factors, including age, ethnicity, current multivitamins use, regular aspirin use, family history of CRC, menopausal status and hormone use (women only) in incidence analysis and additionally for diagnoses of CVD and type 2 diabetes in mortality analysis.

The analyses were conducted using SAS version 9.4 (SAS Institute, Cary, NC). All statistical tests were two sided and P<0.05 was considered statistically significant.

**S1 References**

1. Hu FB, Sigal RJ, Rich-Edwards JW, Colditz GA, Solomon CG, Willett WC, et al. Walking compared with vigorous physical activity and risk of type 2 diabetes in women: a prospective study. Jama. 1999;282(15):1433-9. Epub 1999/10/27. doi: 10.1001/jama.282.15.1433. PubMed PMID: 10535433.

2. Ainsworth BE, Haskell WL, Whitt MC, Irwin ML, Swartz AM, Strath SJ, et al. Compendium of physical activities: an update of activity codes and MET intensities. Med Sci Sports Exerc. 2000;32(9 Suppl):S498-504. Epub 2000/09/19. doi: 10.1097/00005768-200009001-00009. PubMed PMID: 10993420.

3. Pate RR, Pratt M, Blair SN, Haskell WL, Macera CA, Bouchard C, et al. Physical activity and public health. A recommendation from the Centers for Disease Control and Prevention and the American College of Sports Medicine. Jama. 1995;273(5):402-7. Epub 1995/02/01. doi: 10.1001/jama.273.5.402. PubMed PMID: 7823386.

4. van Dam RM, Li T, Spiegelman D, Franco OH, Hu FB. Combined impact of lifestyle factors on mortality: prospective cohort study in US women. BMJ (Clinical research ed). 2008;337:a1440. Epub 2008/09/18. doi: 10.1136/bmj.a1440. PubMed PMID: 18796495; PubMed Central PMCID: PMCPMC2658866.

5. Willett WC, Sampson L, Stampfer MJ, Rosner B, Bain C, Witschi J, et al. Reproducibility and validity of a semiquantitative food frequency questionnaire. American journal of epidemiology. 1985;122(1):51-65. Epub 1985/07/01. doi: 10.1093/oxfordjournals.aje.a114086. PubMed PMID: 4014201.

6. Hu FB, Stampfer MJ, Rimm E, Ascherio A, Rosner BA, Spiegelman D, et al. Dietary fat and coronary heart disease: a comparison of approaches for adjusting for total energy intake and modeling repeated dietary measurements. American journal of epidemiology. 1999;149(6):531-40. Epub 1999/03/20. doi: 10.1093/oxfordjournals.aje.a009849. PubMed PMID: 10084242.

7. World Cancer Research Fund/American Institute for Cancer R. Diet, nutrition, physical activity and cancer: a global perspective. Continuous Update Project Expert Report. 2018.

8. Yu E, Ley SH, Manson JE, Willett W, Satija A, Hu FB, et al. Weight History and All-Cause and Cause-Specific Mortality in Three Prospective Cohort Studies. Annals of internal medicine. 2017;166(9):613-20. Epub 2017/04/07. doi: 10.7326/m16-1390. PubMed PMID: 28384755; PubMed Central PMCID: PMCPMC5518318.

9. Baxter NN, Warren JL, Barrett MJ, Stukel TA, Doria-Rose VP. Association between colonoscopy and colorectal cancer mortality in a US cohort according to site of cancer and colonoscopist specialty. Journal of clinical oncology : official journal of the American Society of Clinical Oncology. 2012;30(21):2664-9. Epub 2012/06/13. doi: 10.1200/jco.2011.40.4772. PubMed PMID: 22689809; PubMed Central PMCID: PMCPMC3413278.

10. Martinez ME, Baron JA, Lieberman DA, Schatzkin A, Lanza E, Winawer SJ, et al. A pooled analysis of advanced colorectal neoplasia diagnoses after colonoscopic polypectomy. Gastroenterology. 2009;136(3):832-41. Epub 2009/01/28. doi: 10.1053/j.gastro.2008.12.007. PubMed PMID: 19171141; PubMed Central PMCID: PMCPMC3685417.
