# Supplementary material for: Healthy lifestyle, endoscopic screening, and colorectal cancer incidence and mortality in the United States: A nationwide cohort study
Source: PLoS Med. 2021 Feb 1;18(2):e1003522. doi: 10.1371/journal.pmed.1003522 (PMC7886195; doi:10.1371/journal.pmed.1003522)
Supplement: Attachment — Submitted filename: Response to reviewers.docx [file pmed.1003522.s017.docx]

We appreciate the opportunity to submit this revised manuscript to *PLOS Medicine*. We have complied with all the publication requirements and addressed the editors’ and reviewers’ comments as detailed below in our point-by-point response. We hope that the revised manuscript will be suitable for publication and we welcome any additional feedback.

In revising the manuscript for further consideration, your revisions should address the specific points made by each reviewer and the editors. Please also check the guidelines for revised papers at [http://journals.plos.org/plosmedicine/s/revising-your-manuscript](https://urldefense.proofpoint.com/v2/url?u=http-3A__journals.plos.org_plosmedicine_s_revising-2Dyour-2Dmanuscript&d=DwMFaQ&c=WO-RGvefibhHBZq3fL85hQ&r=Ht4anKu2fh2hL2Jysum-Lst-Qwp4rhYmu8zDD5_ee_A&m=5vLLs4YuiWlgnC7uVt5eeo05j57izDGgpeKQLByaA3w&s=gxHv6_xAVQC23jcZqFO8Cn8hsHPbUit0XFpLe3UcrVE&e=) for any that apply to your paper. In your rebuttal letter you should indicate your response to the reviewers' and editors' comments, the changes you have made in the manuscript, and include either an excerpt of the revised text or the location (eg: page and line number) where each change can be found. Please submit a clean version of the paper as the main article file; a version with changes marked should be uploaded as a marked up manuscript.

In addition, we request that you upload any figures associated with your paper as individual TIF or EPS files with 300dpi resolution at resubmission; please read our figure guidelines for more information on our requirements: [http://journals.plos.org/plosmedicine/s/figures](https://urldefense.proofpoint.com/v2/url?u=http-3A__journals.plos.org_plosmedicine_s_figures&d=DwMFaQ&c=WO-RGvefibhHBZq3fL85hQ&r=Ht4anKu2fh2hL2Jysum-Lst-Qwp4rhYmu8zDD5_ee_A&m=5vLLs4YuiWlgnC7uVt5eeo05j57izDGgpeKQLByaA3w&s=Tj2zpOnn5qeOKm1ASyglaxku9nMEtIQT0Jg3tLhr9pU&e=). While revising your submission, please upload your figure files to the PACE digital diagnostic tool, [https://pacev2.apexcovantage.com/](https://urldefense.proofpoint.com/v2/url?u=https-3A__pacev2.apexcovantage.com_&d=DwMFaQ&c=WO-RGvefibhHBZq3fL85hQ&r=Ht4anKu2fh2hL2Jysum-Lst-Qwp4rhYmu8zDD5_ee_A&m=5vLLs4YuiWlgnC7uVt5eeo05j57izDGgpeKQLByaA3w&s=c6VpmpyUnL0t0YU56tooSs1ijpiiM8BR2BOyvUtpRWY&e=). PACE helps ensure that figures meet PLOS requirements. To use PACE, you must first register as a user. Then, login and navigate to the UPLOAD tab, where you will find detailed instructions on how to use the tool. If you encounter any issues or have any questions when using PACE, please email us at [PLOSMedicine@plos.org](mailto:PLOSMedicine@plos.org).

We expect to receive your revised manuscript by Sep 17 2020 11:59PM. Please email us ([plosmedicine@plos.org](mailto:plosmedicine@plos.org)) if you have any questions or concerns.

***Please note while forming your response, if your article is accepted, you may have the opportunity to make the peer review history publicly available. The record will include editor decision letters (with reviews) and your responses to reviewer comments. If eligible, we will contact you to opt in or out.***

We ask every co-author listed on the manuscript to fill in a contributing author statement, making sure to declare all competing interests. If any of the co-authors have not filled in the statement, we will remind them to do so when the paper is revised. If all statements are not completed in a timely fashion this could hold up the re-review process. If new competing interests are declared later in the revision process, this may also hold up the submission. Should there be a problem getting one of your co-authors to fill in a statement we will be in contact.  YOU MUST NOT ADD OR REMOVE AUTHORS UNLESS YOU HAVE ALERTED THE EDITOR HANDLING THE MANUSCRIPT TO THE CHANGE AND THEY SPECIFICALLY HAVE AGREED TO IT. You can see our competing interests policy here: [http://journals.plos.org/plosmedicine/s/competing-interests](https://urldefense.proofpoint.com/v2/url?u=http-3A__journals.plos.org_plosmedicine_s_competing-2Dinterests&d=DwMFaQ&c=WO-RGvefibhHBZq3fL85hQ&r=Ht4anKu2fh2hL2Jysum-Lst-Qwp4rhYmu8zDD5_ee_A&m=5vLLs4YuiWlgnC7uVt5eeo05j57izDGgpeKQLByaA3w&s=hY-LUQzfK5V-0FgslvhYM3EYEhIeHyeVAc4XxVX8Myk&e=).

Please use the following link to submit the revised manuscript:

[https://www.editorialmanager.com/pmedicine/](https://urldefense.proofpoint.com/v2/url?u=https-3A__www.editorialmanager.com_pmedicine_&d=DwMFaQ&c=WO-RGvefibhHBZq3fL85hQ&r=Ht4anKu2fh2hL2Jysum-Lst-Qwp4rhYmu8zDD5_ee_A&m=5vLLs4YuiWlgnC7uVt5eeo05j57izDGgpeKQLByaA3w&s=bQ9faXe91JEYpz5-LRFhFH7kUozY5ISyANpC3laga2Q&e=)

Your article can be found in the "Submissions Needing Revision" folder.

To enhance the reproducibility of your results, we recommend that you deposit your laboratory protocols in [protocols.io](https://urldefense.proofpoint.com/v2/url?u=http-3A__protocols.io&d=DwMFaQ&c=WO-RGvefibhHBZq3fL85hQ&r=Ht4anKu2fh2hL2Jysum-Lst-Qwp4rhYmu8zDD5_ee_A&m=5vLLs4YuiWlgnC7uVt5eeo05j57izDGgpeKQLByaA3w&s=e1D9uVvDWIOVe3VnilCuOpHqhJHH40BDkx4d-PYyP74&e=), where a protocol can be assigned its own identifier (DOI) such that it can be cited independently in the future. For instructions see [http://journals.plos.org/plosmedicine/s/submission-guidelines#loc-methods](https://urldefense.proofpoint.com/v2/url?u=http-3A__journals.plos.org_plosmedicine_s_submission-2Dguidelines-23loc-2Dmethods&d=DwMFaQ&c=WO-RGvefibhHBZq3fL85hQ&r=Ht4anKu2fh2hL2Jysum-Lst-Qwp4rhYmu8zDD5_ee_A&m=5vLLs4YuiWlgnC7uVt5eeo05j57izDGgpeKQLByaA3w&s=vgukEHr1k8SeFwCOPou2Xr9XmoJYnuXSrJ_oXjrG4gA&e=).

Please ensure that the paper adheres to the PLOS Data Availability Policy (see [http://journals.plos.org/plosmedicine/s/data-availability](https://urldefense.proofpoint.com/v2/url?u=http-3A__journals.plos.org_plosmedicine_s_data-2Davailability&d=DwMFaQ&c=WO-RGvefibhHBZq3fL85hQ&r=Ht4anKu2fh2hL2Jysum-Lst-Qwp4rhYmu8zDD5_ee_A&m=5vLLs4YuiWlgnC7uVt5eeo05j57izDGgpeKQLByaA3w&s=23BPD3eFFifMQqt3wV8yiV7AwC_HohsXUcbBo-1L_-Y&e=)), which requires that all data underlying the study's findings be provided in a repository or as Supporting Information. For data residing with a third party, authors are required to provide instructions with contact information for obtaining the data. PLOS journals do not allow statements supported by "data not shown" or "unpublished results." For such statements, authors must provide supporting data or cite public sources that include it.

We look forward to receiving your revised manuscript.

Sincerely,

Emma Veitch, PhD

PLOS Medicin
On behalf of Clare Stone, PhD, Acting Chief Editor,
PLOS Medicine
[plosmedicine.org](https://urldefense.proofpoint.com/v2/url?u=http-3A__plosmedicine.org&d=DwMFaQ&c=WO-RGvefibhHBZq3fL85hQ&r=Ht4anKu2fh2hL2Jysum-Lst-Qwp4rhYmu8zDD5_ee_A&m=5vLLs4YuiWlgnC7uVt5eeo05j57izDGgpeKQLByaA3w&s=lPidYKNSpuzrUqBoIsRgmvi5IVG48mF4vFjp3ylz2vI&e=)

-----------------------------------------------------------

Requests from the editors:

1. *Please structure your abstract using the PLOS Medicine headings (Background, Methods and Findings, Conclusions - "methods and findings" is a single subsection).

Thank you for four comments. We have addressed this by combining the Methods and Findings in a single subsection.

2. *In the last sentence of the Abstract Methods and Findings section, please describe the main limitation(s) of the study's methodology.

We have addressed this by adding a main limitation in the last sentence of the Methods and Findings section.

(Abstract, Page 2)

The corresponding PAR (95% CI) increased from 15% (13-16%) to 51% (17-74%) for proximal colon cancer and from 47% (45-50%) to 75% (61-84%) for distal CRC. Results were similar for CRC mortality. A limitation of our study is that our study participants are all health professionals and predominantly whites, which may not be representative of the general population.

3. *In the abstract conclusions, the text currently reads "Healthy lifestyle may reduce CRC incidence and mortality independent of endoscopic screening", but this assumes that the effect sizes seen in the main analyses represent causal effects, and given the study design we can't assume this. It would be better in the abstract to use more moderate language and to present the findings consistently as associations - in the main discussion you can then go on to say that, if those effects were causal then the implications might mean (xyz...), but this conclusion shouldn't be leapt to.

We have revised the abstract conclusions to avoid this confusion.

(Abstract, Page 3)

**Conclusions:** Healthy lifestyle may be associated with lower CRC incidence and mortality independent of endoscopic screening. An integration of healthy lifestyle with endoscopic screening may substantially enhance prevention for CRC, particularly for proximal colon cancer, compared to endoscopic screening alone.

We have also revised this kind of statements throughout the main text.

(Discussion, Page 15)

If these associations were causal, approximately 32% of CRC cases and 34% of CRC deaths could potentially be prevented by endoscopic screening alone and ….

(Discussion, Page 16)

If these effects were causal, our findings have important clinical and public health implications. First, we provide empirical evidence for the independent associations of healthy lifestyle and endoscopic screening with lower CRC incidence and mortality.

(Discussion, Page 18)

In conclusion, …. If these effects were causal, integration of healthy lifestyle with endoscopic screening may improve prevention of CRC, particularly proximal colon cancer, compared to endoscopic screening alone.

4. *Please use the following style for the author summary - [https://journals.plos.org/plosmedicine/s/revising-your-manuscript#loc-author-summary](https://urldefense.proofpoint.com/v2/url?u=https-3A__journals.plos.org_plosmedicine_s_revising-2Dyour-2Dmanuscript-23loc-2Dauthor-2Dsummary&d=DwMFaQ&c=WO-RGvefibhHBZq3fL85hQ&r=Ht4anKu2fh2hL2Jysum-Lst-Qwp4rhYmu8zDD5_ee_A&m=5vLLs4YuiWlgnC7uVt5eeo05j57izDGgpeKQLByaA3w&s=n0OzrtL1IWE_27HEHGl9V-IOzDh2irtCX-BEY1AZIIg&e=) - this should use bullets and the headers need to be different. See also an example, [https://journals.plos.org/plosmedicine/article?id=10.1371/journal.pmed.1002416](https://urldefense.proofpoint.com/v2/url?u=https-3A__journals.plos.org_plosmedicine_article-3Fid-3D10.1371_journal.pmed.1002416&d=DwMFaQ&c=WO-RGvefibhHBZq3fL85hQ&r=Ht4anKu2fh2hL2Jysum-Lst-Qwp4rhYmu8zDD5_ee_A&m=5vLLs4YuiWlgnC7uVt5eeo05j57izDGgpeKQLByaA3w&s=k02HbBnJxbWFpUCPiZWQ9ZB7M5CLr5x-RJ1V9Vpu_Xc&e=)

We have revised the Author Summary according to the guideline and the provided example.

(Author Summary, Pages 4-5)

Author Summary

**Why was this study done?**

- Colorectal cancer is the third leading cause of cancer death in the US.
- Healthy lifestyle and screening represent two major approaches to colorectal cancer prevention.
- It remains unknown whether individuals having undergone endoscopic screening can still benefit from healthy lifestyle.
- It remains to be determined to what extent a combination of healthy lifestyle and endoscopic screening can improve colorectal cancer prevention compared with endoscopic screening alone.

**What did the researchers do and find?**

- Using two large nationwide cohorts in the US with a median of 26 years of follow-up, we prospectively examined the association of healthy lifestyle with colorectal cancer incidence and mortality among individuals having and not having undergone endoscopic screening separately.
- We also estimated the proportion of colorectal cancer cases and deaths that are potentially prevented by endoscopic screening alone and by a combination of healthy lifestyle and endoscopic screening, assuming the effects are causal.
- We found a similar association between healthy lifestyle and lower incidence and mortality of colorectal cancer among individuals having and not having undergone endoscopic screening.
- We also found that an integration of healthy lifestyle with endoscopic screening might substantially enhance the prevention of colorectal cancer and related death compared with endoscopic screening alone, and the incremental benefit was particularly strong for proximal colon cancer.

**What do these findings mean?**

- Healthy lifestyle is associated with lower colorectal cancer incidence and mortality independent of endoscopic screening status.
- The evidence highlights the potential of healthy lifestyle to complement endoscopic screening for optimal prevention of colorectal cancer.
- Our findings highlight the particular merit of an integration of healthy lifestyle and endoscopic screening for prevention of proximal colon cancer, for which endoscopic screening has limited effectiveness.
- These findings suggest the value of healthy lifestyle for colorectal cancer prevention in settings lacking coverage or capacity of endoscopic screening.

5. *We'd suggest ensuring that the study is reported according to the STROBE guideline; when doing so, please include the completed STROBE checklist as Supporting Information. Please add the following statement, or similar, to the Methods: "This study is reported as per the Strengthening the Reporting of Observational Studies in Epidemiology (STROBE) guideline (S1 Checklist)." The STROBE guideline can be found here: [http://www.equator-network.org/reporting-guidelines/strobe/](https://urldefense.proofpoint.com/v2/url?u=http-3A__www.equator-2Dnetwork.org_reporting-2Dguidelines_strobe_&d=DwMFaQ&c=WO-RGvefibhHBZq3fL85hQ&r=Ht4anKu2fh2hL2Jysum-Lst-Qwp4rhYmu8zDD5_ee_A&m=5vLLs4YuiWlgnC7uVt5eeo05j57izDGgpeKQLByaA3w&s=MxfwAweBorxDW-KLhR7MFrGoT9F7xmwMhwrdvguHP6w&e=). When completing the checklist, please use section and paragraph numbers, rather than page numbers.

We have completed the STROBE checklist and uploaded it as a Supporting Information file (S1 checklist).

Also, we have added the statement in the Methods:

(Methods, Page 7)

The study protocol was approved by the institutional review board of the Brigham and Women’s Hospital and Harvard T.H. Chan School of Public Health, and those of participating registries as required. This study was reported as per the Strengthening the Reporting of Observational Studies in Epidemiology (STROBE) guideline (S1 Checklist).

6. *Did your study have a prospective protocol or analysis plan? Please state this (either way) early in the Methods section.

   a) If a prospective analysis plan (from your funding proposal, IRB or other ethics committee submission, study protocol, or other planning document written before analyzing the data) was used in designing the study, please include the relevant prospectively written document with your revised manuscript as a Supporting Information file to be published alongside your study, and cite it in the Methods section. A legend for this file should be included at the end of your manuscript.

We did have a prospective analysis proposal and have uploaded it as a Supporting Information file (S1 Text).

Also, we have cited the prospective analysis proposal in the Methods section:

(Methods, Page 7)

We conducted this study following a prospective analysis proposal (S1 Text). The study protocol was approved by the institutional review board of the Brigham and Women’s Hospital and Harvard T.H. Chan School of Public Health, and those of participating registries as required.

We have also included a legend for the prospective analysis proposal at the end of the manuscript.

(Legends of Supporting Information, Page 28)

S1 Text. Prospective analysis proposal.

b) If no such document exists, please make sure that the Methods section transparently describes when analyses were planned, and when/why any data-driven changes to analyses took place.  

c) In either case, changes in the analysis-- including those made in response to peer review comments-- should be identified as such in the Methods section of the paper, with rationale.

7. *In the discussion section, as noted above for the abstract, some of the language should be moderated so it doesn't assume causal effects. Eg, "Approximately 32% of CRC cases and 34% of CRC deaths could potentially be prevented by endoscopic screening alone" - this could be presented later, but with the caveat that these estimates are based on assuming the effect size seen in your analyses are entirely reflective of a "true" causal effect (which may not be true). The discussion in general should be careful about where it uses causal language and it would be better to ensure the main part of the discussion presents the findings as associations, only going on later to discuss the implications **if these associations are indeed causal**.

As we respond to the Comment 3, we have revised all the “causal language” throughout the Abstract and main text. Details of the revisions please see our response to the Comment 3.

-----------------------------------------------------------

Comments from the reviewers:

Reviewer #1: I confine my remarks to statistical aspects of this paper.  The general approach is OK but I have a number of issues to resolve before I can recommend publication.

8. First, quoting a "per unit" estimate is misleading, as the trend in both groups was non-linear (and nonlinear in different ways).

Thank you for your comments. We agree that quoting a “per unit” estimate can be misleading if the linearity assumption does not hold for the relationship between the lifestyle score and outcome. To test linearity, we have added a restricted cubic spline analysis in the Cox regression model to examine any possible non-linear relation between lifestyle score and incidence and mortality of colorectal cancer. This analysis reported P values for both non-linearity and linearity. We have added the statement and rationale of this analysis in the Statistical Analysis section and added the analysis results in the Results section and Supporting Information (S2 Fig).

(Methods, Page 10)

… for the associations of individual and combined lifestyle factors with CRC incidence and mortality according to endoscopic screening status. We applied restricted cubic spline analysis in the Cox regression model [26] to examine whether there is a linear relationship between the healthy lifestyle score and CRC incidence and mortality.

Reference:

26. Desquilbet L, Mariotti F. Dose-response analyses using restricted cubic spline functions in public health research. Statistics in medicine. 2010;29(9):1037-57. Epub 2010/01/21. doi: 10.1002/sim.3841. PubMed PMID: 20087875.

(Results, Page 12)

Figure 1 shows the associations of healthy lifestyle score with CRC incidence and mortality according to endoscopic screening status. The spline analysis indicated that the relationships were linear (S2 Fig).

(Supporting Information, S2 Fig)

**S2 Fig. Spline curves from restricted cubic spline analysis in Cox proportional hazards regression models to examine the possibly non-linear relationship between the healthy lifestyle score and colorectal cancer (CRC) incidence and mortality according to endoscopic screening status**. The analysis was adjusting for age, calendar period, sex, ethnicity, current multivitamin use, regular aspirin use, family history of colorectal cancer, menopausal status and hormone use (women only) in incidence analysis and additionally diagnoses of cardiovascular disease and type 2 diabetes in mortality analysis. In both the unscreened and screened groups, no spline variables were selected into the models for CRC incidence and mortality, indicating the linearities hold (incidence: unscreened P<0.001, screened P<0.001; mortality: unscreened P=0.005, screened: P=0.004).

9. Second, the interaction term added is not adequate, as it does not address the above. Consider CRC incidence (as shown in fig 1A). If the factor of 0.85 was correct, then the nonscreened group would have HRs of 1, 0.85, 0.72, 0.61, 0.52 and 0.44; while the screened group would have values of 0.37 0.31, 0.27 0.23 0.19 amd 0.16.  These are not accurate (and the two lines are not parallel)

Although the point estimates of the HRs seemed to have a non-linear trend, as responded above, formal statistical testing using the spline analysis did not show any significant non-linear relationship between the lifestyle score and outcome. Moreover, as shown in the spline figure, the confidence intervals in the two groups largely overlap, consistent with our findings for no statistically significant interactions. Therefore, we believe that the interaction analysis using the product term is an appropriate approach in this setting.

10. Third, it's an interesting question whether the appropriate measure for reduction in risk is the ratio or the difference. Reducing a large risk by (say) 15% is not the same as reducing a small risk by 15%.

We agree that the difference in the risk (Absolute Risk Reduction, AAR) is also an important measure for risk reduction. To highlight this, we presented the absolute risks (incidence rate and mortality rate) according to the healthy lifestyle score and endoscopic screening status in the secondary y-axis in Fig 1. We hope this could complement the hazard ratios to comprehensively represent the reduced risk.

11. More specific points: On p. 5 at the top, give a rate of CRC death per person, not just "3rd highest"

We have added the rates of CRC incidence and mortality.

(Introduction, Page 6)

Colorectal cancer (CRC) is the third most commonly diagnosed cancer (40.7 per 100,000 population) and the third leading cause of cancer death (14.8 per 100,000 population) in the United States (U.S.) according to the estimates in 2020 [1].

Reference

1. Siegel RL, Miller KD, Jemal A. Cancer statistics, 2020. CA: a cancer journal for clinicians. 2020;70(1):7-30.

12. Also on p. 5, the huge range of risk reduction for liefestyle (20 to 70%) makes these claims less credible (at least to me).

We agree with this comment. Through a detailed look into the literature, this wide range (20-70%) may be due to a number of factors across the studies, e.g., they might not include exactly the same lifestyle factors in the definition of healthy lifestyle; they might use different low-risk criteria for the same lifestyle factor; they might use different methods in the calculation of the population attributable risks. We are conducting a separate study to empirically investigate the reasons for the variation.

13. p. 7 Please define "metabolic equivalents"

We have added the definition of "metabolic equivalents" in the Supporting Information (S2 Text).

(Supporting Information, S2 Text)

We assessed physical activity biennially by a validated questionnaire and calculated the total hours per week for moderate-to-vigorous intensity activity (including brisk walking) that requires the expenditure of at least 3 metabolic equivalents (METs) per hour. A MET is the ratio of work metabolic rate to a standard resting metabolic rate of 1.0 (4.184 kJ) kg^−1^ hr^−1^ [2]. One MET is the resting metabolic rate while sitting [2]; activities between 3 and 6 METs are considered moderate intensity, while activities >6 METs are considered vigorous intensity [3].

S1 References:

2. Ainsworth BE, Haskell WL, Whitt MC, Irwin ML, Swartz AM, Strath SJ, et al. Compendium of physical activities: an update of activity codes and MET intensities. Med Sci Sports Exerc. 2000;32(9 Suppl):S498-504. Epub 2000/09/19. doi: 10.1097/00005768-200009001-00009. PubMed PMID: 10993420.

3. Pate RR, Pratt M, Blair SN, Haskell WL, Macera CA, Bouchard C, et al. Physical activity and public health. A recommendation from the Centers for Disease Control and Prevention and the American College of Sports Medicine. Jama. 1995;273(5):402-7. Epub 1995/02/01. doi: 10.1001/jama.273.5.402. PubMed PMID: 7823386.

14. p. 7  I'm not altogether happy with the way the lifestyle factor was computed. Dichotomoizing risk factors is kind of silly. People don't go from "normal" to "high" risk when they cross some threshold. E.g., for BMI (a very flawed measure of obesity) you aren't fine at 24.9 and then high risk at 25.1.  Also, 31 minutes of exercise is not dramatically different from 29 minutes. It would be better to develop a measure of risk that left all measures continuouis (and maybe nonllinear).eith based on data or based on literature.

We agree that for a dichotomized continuous variable, there is no essential difference between values that are a little bit smaller or larger than the dichotomizing criterion. But in our study, we created the healthy lifestyle score by dichotomizing each of the lifestyle factors based on a prior knowledge of their associations with CRC and available national and international public health recommendations, some examples of the literature are:

- Aune, D., Chan, D. S., Lau, R., Vieira, R., Greenwood, D. C., Kampman, E., & Norat, T. (2011). Dietary fibre, whole grains, and risk of colorectal cancer: systematic review and dose-response meta-analysis of prospective studies. Bmj, 343.
- Liang, P. S., Chen, T. Y., & Giovannucci, E. (2009). Cigarette smoking and colorectal cancer incidence and mortality: Systematic review and meta‐analysis. International journal of cancer, 124(10), 2406-2415.
- Bagnardi, V., Rota, M., Botteri, E., Tramacere, I., Islami, F., Fedirko, V., ... & Pelucchi, C. (2015). Alcohol consumption and site-specific cancer risk: a comprehensive dose–response meta-analysis. British journal of cancer, 112(3), 580-593.
- Boyle, T., Keegel, T., Bull, F., Heyworth, J., & Fritschi, L. (2012). Physical activity and risks of proximal and distal colon cancers: a systematic review and meta-analysis. Journal of the national cancer institute, 104(20), 1548-1561.
- Ma, Y., Yang, Y., Wang, F., Zhang, P., Shi, C., Zou, Y., & Qin, H. (2013). Obesity and risk of colorectal cancer: a systematic review of prospective studies. PloS one, 8(1), e53916.

Also, the healthy lifestyle score created by the dichotomized lifestyle factors has been widely used by us before, e.g.,

- Song, M., & Giovannucci, E. (2016). Preventable incidence and mortality of carcinoma associated with lifestyle factors among white adults in the United States. JAMA oncology, 2(9), 1154-1161.
- Li, Yanping, et al (2018). Impact of healthy lifestyle factors on life expectancies in the US population. Circulation, 138 (4): 345-355.

and also by other groups, e.g.,

- Kirkegaard, H., Johnsen, N. F., Christensen, J., Frederiksen, K., Overvad, K., & Tjønneland, A. (2010). Association of adherence to lifestyle recommendations and risk of colorectal cancer: a prospective Danish cohort study. Bmj, 341, c5504.
- Carr, P. R., Weigl, K., Jansen, L., Walter, V., Erben, V., Chang-Claude, J., ... & Hoffmeister, M. (2018). Healthy lifestyle factors associated with lower risk of colorectal cancer irrespective of genetic risk. Gastroenterology, 155(6), 1805-1815.

In addition, we think the dichotomization in our study simplifies 1) the interpretation of our findings, i.e., one-unit increase in the score represents improvement in one lifestyle factor; and 2) the public health message for future translation.

However, to address the reviewer’s concern, we have added a sensitivity analysis by using the 5 lifestyle factors as 5-categorical variables:

- BMI: 18.5-24.9, 25.0-27.4, 27.5-29.5, 30.0-34.9, and ≥35.0 kg/m^2^ (scored 5-1)
- Smoking: never, past smoker with <5 pack-years, past smoker with ≥5 pack-years, current smoker with <20 pack-years, current smoker with ≥20 pack-years (scored 5-1)
- Alcohol intake: 0, 0.1-13.9, 14-20.9, 21-27.9, and ≥28 g/d (scored 5-1)
- Physical activity: 0, 0.1-0.9, 1.0-3.4, 3.5-5.9, and ≥6 h/week (scored 1-5)
- Number of healthy dietary components: 0-1, 2, 3, 4, and 5-6 (scored 1-5)

Sum of the 5 scores (rang, 5-25) was then categorical into 5 levels (5-10, 11-13, 14-16, 17-19, 20-22, and 23-25). These 5 levels were defined as the new healthy lifestyle score (0, 1, 2, 3, 4, and 5). Using this new lifestyle score, we calculated the hazard ratios (HRs) and population attributable risks (PARs) in Supporting Information (S6 Fig and S8 Table). The results were essentially unchanged compared to our primary analysis.

(Methods, Pages 11-12)

In the main analysis, we counted the cycles when no response to endoscopy was recorded (accounting for 11% of all person-time) into the non-screening group, so we repeated the analysis after excluding the cycles as a sensitivity analysis. Because the binary variables could not account for the gradient in CRC risk with more extreme levels of these lifestyle factors, we conducted another sensitivity analysis by calculating an expanded healthy lifestyle score using more refined categorizations for the lifestyle factors. We assigned scores of 1 (least healthy) to 5 (most healthy) to the categories of the lifestyle factors and summed the scores across all 5 factors (range, 5–25). We then categorized this expanded score into 6 levels (5-10, 11-13, 14-16, 17-19, 20-22, and 23-25), which were defined as the new healthy lifestyle score (0, 1, 2, 3, 4, and 5).

(Results, Pages 14-15)

Our results were robust to several sensitivity analyses, including … excluding the person-times when response to endoscopy was missing (S7 Table), and defining the healthy lifestyle score using the 5 lifestyle factors as 5-categorical variables (S6 Fig and S8 Table).

(Supporting Information, S6 Fig)

**S6 Fig. Association of healthy lifestyle score with colorectal cancer incidence according to endoscopic screening status when healthy lifestyle score was defined by the 5 5-catgorocal lifestyle factors.** The 5 5-categorical lifestyle factors included body mass index (18.5-24.9, 25.0-27.4, 27.5-29.5, 30.0-34.9, and ≥35.0 kg/m^2^; scored 5-1, respectively), smoking (never, past smoking with pack-years <5, past smoking with pack-years ≥5, current smoker with pack-years <20, and current smoker with pack-years ≥20; scored 5-1, respectively), physical activity (moderate-to-vigorous intensity activity for 0, 0.1-0.9, 1.0-3.4, 3.5-5.9, and ≥6 hours per week; scored 1-5, respectively), alcohol intake (0, 0.1-13.9, 14-20.9, 21-27.9, and ≥28 g/d; scored 5-1, respectively), and number of the 6 healthy dietary components recommendations by the World Cancer Research Fund/American Institute for Cancer Research Third Expert Report 2018 (0-1, 2, 3, 4, and 5-6; scored 1-5, respectively). The 6 healthy dietary components included red meat <0.5 serving/d, processed meat <0.2 serving/d, dietary fiber ≥30 g/d, dairy products ≥3 servings/d, whole grains ≥48 g/d or account for at least half of total grains, and calcium supplement use. Sum of the 5 scores (rang, 5-25) was then categorical into 5 levels (5-10, 11-13, 14-16, 17-19, 20-22, and 23-25). These 5 levels were defined as the new healthy lifestyle score (0, 1, 2, 3, 4, and 5). Multivariable Cox proportional hazards regression was used to calculate the hazard ratios (HRs) and 95% confidence intervals (CIs) while adjusting for age, calendar period, sex, ethnicity, current multivitamin use, regular aspirin use, family history of colorectal cancer, menopausal status and hormone use (women only). Error bars indicate 95% CIs.

(Supporting Information, S8 Table)

**S8 Table. Population-attributable risk estimates for colorectal cancer incidence with endoscopic screening and healthy lifestyle separately and in combination when healthy lifestyle score was defined by the 5 5-catgorocal lifestyle factors^*^**

| Exposures | % PAR (95% CI)^†^ |
| --- | --- |
| Endoscopic screening | 32 (31-33) |
| Healthy lifestyle score ≥2 | 6 (4-7) |
| Healthy lifestyle score ≥3 | 15 (11-19) |
| Healthy lifestyle score ≥4 | 23 (14-31) |
| Healthy lifestyle score =5 | 38 (9-62) |
| Endoscopic screening & healthy lifestyle score ≥2 | 36 (33-39) |
| Endoscopic screening & healthy lifestyle score ≥3 | 42 (37-47) |
| Endoscopic screening & healthy lifestyle score ≥4 | 48 (41-55) |
| Endoscopic screening & healthy lifestyle score =5 | 60 (38-74) |

Abbreviations: PAR, population-attributable risk; CI, confidence interval.

^*^The 5 5-categorical lifestyle factors included body mass index (18.5-24.9, 25.0-27.4, 27.5-29.5, 30.0-34.9, and ≥35.0 kg/m^2^; scored 5-1, respectively), smoking (never, past smoking with pack-years <5, past smoking with pack-years ≥5, current smoker with pack-years <20, and current smoker with pack-years ≥20; scored 5-1, respectively), physical activity (moderate-to-vigorous intensity activity for 0, 0.1-0.9, 1.0-3.4, 3.5-5.9, and ≥6 hours per week; scored 1-5, respectively), alcohol intake (0, 0.1-13.9, 14-20.9, 21-27.9, and ≥28 g/d; scored 5-1, respectively), and number of the 6 healthy dietary components recommendations by the World Cancer Research Fund/American Institute for Cancer Research Third Expert Report 2018 (0-1, 2, 3, 4, and 5-6; scored 1-5, respectively). The 6 healthy dietary components included red meat <0.5 serving/d, processed meat <0.2 serving/d, dietary fiber ≥30 g/d, dairy products ≥3 servings/d, whole grains ≥48 g/d or account for at least half of total grains, and calcium supplement use. Sum of the 5 scores (rang, 5-25) was then categorical into 5 levels (5-10, 11-13, 14-16, 17-19, 20-22, and 23-25). These 5 levels were defined as the new healthy lifestyle score (0, 1, 2, 3, 4, and 5).

^†^PARs and 95% CIs were calculated while adjusting for age, calendar period, sex, ethnicity, current multivitamin use, regular aspirin use, family history of colorectal cancer, menopausal status and hormone use (women only).

15. p 10  How was the "per unit" number calculated, given that there were 5 different logistic regressions run for each group?

    As stated in the Methods “Assessment of Lifestyle Factors”, an overall healthy lifestyle score (range, 0-5) was calculated by summing the scores of the 5 individual lifestyle factors. Thus, the HRs associated with each unit increment in the healthy lifestyle score were estimated by treating the score as a continuous variable in the Cox proportional hazards regression model. This was to examine the overall trend of the association between the healthy lifestyle score and CRC incidence and mortality.

----------------------------------------------------------

Reviewer #2: Wang and colleagues address an important topic- the associations between screening and/or  healthy lifestyles and the risk of colorectal cancer incidence and mortality, using the Nurses health Study and Health professionals follow-up study, two large, important cohorts that have been sources for many important observational studies. They found that the protective effects of screening and health lifestyle (based on a healthy lifestyle score) were independently associated with lower risks of CRC incidence and mortality. These findings are important and timely. The study is well-executed and the supplementary analyses helpful. I would make a few suggestions to better put them in context:

16. 1) I would ask that the language be changed to highlight these are associations, not interventions or trials: For example, instead of concluding: "Healthy lifestyle may reduce CRC incidence and mortality independent of endoscopic screening." I would suggest "Healthy lifestyle is associated with lower CRC incidence and mortality independent of endoscopic screening." Similarly, saying "First, we provide empirical evidence for the substantial benefit of lifestyle modification, independent of endoscopic screening, for improved CRC prevention." is probably less accurate than "First, we provide empirical evidence for the independent association of healthy behaviors and endoscopic screening with lower CRC incidence."

Thank you for your comments. As responded to the Comment 3, we have revised these statements throughout the Abstract and main text.

17. 2) It would be helpful to discuss the rationale for turning the actual, sometimes linear measures included in the healthy lifestyle score into dichotomized scores. Giving the same weight to 3 drinks per day and 10 drinks per day, for example, seems to underweight extreme scores.

Please see our response to the Comment 14.

18. 3) What is the relationship between the lifestyle score and all-cause mortality? I am assuming the the effects go beyond that of CRC.

The lifestyle score has a well-documented association with lower all-cause mortality. This relationship has been reported both in our cohorts:

- Li, Yanping, et al (2018). Impact of healthy lifestyle factors on life expectancies in the US population. Circulation, 138 (4): 345-355

and by other groups:

- Ford, E. S., Bergmann, M. M., Boeing, H., Li, C., & Capewell, S. (2012). Healthy lifestyle behaviors and all-cause mortality among adults in the United States. Preventive medicine, 55(1), 23-27.

19. 4) The discussion talks about "lifestyle modification" which is an intervention framing, but I think it is important to reiterate the associations are with given levels of healthy behaviors moreso than the results of chnages in those behaviors. This framing is important because while both screening and healthy lifestyles are associated with less incidence and mortality, the effort required to achieve the protective levels of lifestyle (medium to high intensity counseling interventions) may be considerably greater than those required to get endoscopic screening (a discrete event).

We agree that we should avoid the statements that are like an intervention study because our current study is looking at the long-term overall pattern in lifestyle rather than change over time. Thus, in addition to the revises made as we responded to the Comment 3, we have revised all the “lifestyle modification” throughout the manuscript to be “healthy lifestyle”.

Also, we agree that the efforts required for healthy lifestyle adoption and endoscopic screening are likely different to reach a similar level of protection against CRC. However, our study aimed to examine how much healthy lifestyle could add on the top of endoscopic screening rather than to replace endoscopic screening. In the Discussion, we have highlighted this point.

(Discussion, Page 17)

We found that each increment in the number of healthy lifestyle practices was associated with 14-17% reduction in mortality due to other causes than CRC. However, no such benefit was observed for endoscopic screening. Nevertheless, given the challenges in undertaking and maintaining healthy lifestyle practices (only 3% of our study population had all five healthy lifestyle factors), screening should still serve as an unshakable cornerstone for CRC prevention.

-----------------------------------------------------------

Reviewer #3: From non-randomized studies on colonoscopy screening as well as from randomized studies on sigmoidoscopy and FOBT studies we have learned that endoscopic screening is a sufficient tool to reduce incidence and mortality from colorectal cancer. Furthermore, epidemiologigal studies have identified various life style associated risk factors for colorectal cancer, including BMI, smoking, nutrition. Probably up to 50% of colorectal cancers could be prevented by a healthy life style. Whereas both a healthy life style and screening can reduce the colorectal cancer burden and both are recommended by various national and international guidelines, it is so far not known whether individuals undergoing screening colonoscopy can further reduce their colorectal cancer risk by maintaining a healthy life style.
Wang et al. provide an answer to this important question in their study. Based on the analysis of well-known and thoroughly studied cohorts, the nurses health study and the health professionals follow-up study, they are able to demonstrate that the reduction of colorectal cancer incidence and mortality by maintaining a healthy life style is independent of participating in colonoscopy screening. Furthermore, individuals who undergo colonoscopy and follow a healthy life style are able to further reduce their colon cancer risk. Therefore, the recommendation will be to participate in bowel screening and to maintain a healthy lifestyle in oder to minimize risk. To the best of my knowledge, this is the first study to provide this evidence.
The study is well designed, the analyzes have been properly performed and the manuscipt is well written. I only have some minor issues:

20. 1.      Is there data available on the adenomatous polyp count / size in the colonoscopies? If yes, is there a difference regarding life style? I would expect that a healthy life style is associated with a lower polyp count.

Thank you for your comments. Yes, we have comprehensive data on polyp counts and sizes and have reported the strong associations of lifestyle factors with polyps in a recent publication:

- He, X., Wu, K., Ogino, S., Giovannucci, E. L., Chan, A. T., & Song, M. (2018). Association between risk factors for colorectal cancer and risk of serrated polyps and conventional adenomas. Gastroenterology, 155(2), 355-373

21. 2.      Were the colonoscopies primary screening colonoscopies? Or did some patients have sigmoidoscopies or FOB testing before with a finding that resulted in referral to secondary screening by colonoscopy? How did you include negative screening by sigmoidoscopy or negative FOBT screening in your analysis? Somebody having negative sigmoidoscopy and/or negative FOBT every other year has undergone screening that will also reduce the risk of a diagnosis of colorectal cancer and will usually not undergo colonoscopy. If data on FOBT screening is available, data for sigmoidoscopies is obviously available, it would be important to study the group with no screening at all as the unscreened group currently comprises no screeing at all + screening other than colonoscopy.

We agree that a person having negative sigmoidoscopy or fecal occult blood test (FOBT) will usually not undergo colonoscopy screening within the next couple of years. To rule out the effect of negative sigmoidoscopy, we performed a sensitivity analysis by excluding sigmoidoscopy and only focusing on colonoscopy screening (S5 Table). We observed no considerable difference from the current main results.

However, we do not have the data on FOBT and have acknowledged this as a limitation in the Discussion section.

(Discussion, Page 18)

On the other hand, given the generally healthier lifestyle profiles of health professionals, we may have underestimated the benefit of lifestyle in the general population. Additionally, we lacked data on other screening modalities than endoscopy and were thus unable to examine their influence on our results.

22. 3.      How did authors account for changes in life style, e.g. unhealthy lifestyle in the beginning of the study and changing to a healthier diet over time?

We accounted for changes in lifestyles by using time-varying exposures in the analysis. We have added more details to the methods.

(Methods, Page 10)

We first calculated the age-adjusted CRC incidence and mortality rates by healthy lifestyle score and endoscopic screening status. To account for multiple records per participant and time-varying exposures and covariates, we used an Andersen-Gill data structure with a new record for each 2-year follow-up period. Then we used time-varying and age-, period-, and cohort-stratified Cox proportional hazards regression models to estimate the multivariable hazard ratios (HRs) and 95% confidence intervals (CIs) for the associations of individual and combined lifestyle factors with CRC incidence and mortality according to endoscopic screening status.

23. 4.      Was there a difference in Duke`s or UICC staging of the cancers diagnosed between groups? As a stage shift towards lower stages by screening is known, it could be speculated that the group with screening + healthy diet not only has less cancers but also cancers at earlier stages.

We agree that analysis according to CRC stage is interesting and important. We have added a calculation of HRs and PARs stratified by CRC TNM stage (TNM 1&2 and 3&4) in the main results.

(Methods, Page 10)

… those occurring in the splenic flexure, descending colon, sigmoid colon, rectosigmoid junction, and rectum. We also staged CRC according to the American Joint Committee on Cancer (AJCC) tumor–node–metastasis (TNM) cancer staging system [25].

Reference:

25. Edge SB, Compton CC. The American Joint Committee on Cancer: the 7th edition of the AJCC cancer staging manual and the future of TNM. Ann Surg Oncol. 2010;17(6):1471-4. Epub 2010/02/25. doi: 10.1245/s10434-010-0985-4. PubMed PMID: 20180029.

(Methods, Page 10)

We also assessed the associations by anatomical site (proximal colon cancer or distal CRC) and AJCC TNM stage of CRC (TNM 1&2 or 3&4) using the subtype-stratified Cox ….

(Results, Page 13)

When assessed by anatomical site and TNM stage, the associations remained similar between the screening and non-screening groups for proximal colon cancer (P-interaction =0.29) and distal CRC (P-interaction =0.93) (S3 Fig), and TNM 1&2 (P-interaction =0.49) and TNM 3&4 CRC (P-interaction =0.86) (S4 Fig).

(Results, Page 14)

We also calculated the PARs by CRC subsites (Fig 2) and TNM stage (Fig 3). … The corresponding PAR for distal CRC increased from 47% (95% CI, 45-50%) to 75% (95% CI, 61-84%), for TNM 1&2 CRC from 27% (95% CI, 26-28%) to 52% (95% CI, 31-68%), and for TNM 3&4 CRC from 37% (95% CI, 33-41%) to 68% (95% CI, 45-84%).

(Results, Fig 3)

**Fig 3. Population-attributable risk (PAR) estimates for incidence of colorectal cancer by TNM stage (TNM 1&2 and 3&4) with endoscopic screening alone and endoscopic screening-healthy lifestyle combination**. Healthy lifestyle score (range, 0-5) was defined as the number of the 5 healthy lifestyle factors: normal body weight (body mass index, ≥18.5 and <25.0 kg/m2), never smoking or past smoking with pack-years <5, moderate-to-vigorous intensity activity for ≥30 minutes per day, none-to-moderate alcohol intake (<1 drink [14g alcohol]/d for women and <2 drinks/d for men), and meeting at least 3 of the 6 dietary recommendations by the World Cancer Research Fund/American Institute for Cancer Research Third Expert Report 2018, which included red meat <0.5 serving/d, processed meat <0.2 serving/d, dietary fiber ≥30 g/d, dairy products ≥3 servings/d, whole grains ≥48 g/d or account for at least half of total grains, and calcium supplement use. PARs and 95% confidence intervals (CIs) were calculated while adjusting for age, calendar period, sex, ethnicity, current multivitamin use, regular aspirin use, family history of colorectal cancer, menopausal status and hormone use (women only). Error bars indicate 95% CIs.

(Supporting Information, S4 Fig)

**S4 Fig. Association of healthy lifestyle score with incidence of colorectal cancer by TNM stage (panel A: TNM 1&2; panel B: TNM 3&4) according to endoscopic screening status**. Healthy lifestyle score (range, 0-5) was defined as the number of the 5 healthy lifestyle factors: normal body weight (body mass index, ≥18.5 and <25.0 kg/m^2^), never smoking or past smoking with pack-years <5, moderate-to-vigorous intensity activity for ≥30 minutes per day, none-to-moderate alcohol intake (<1 drink [14g alcohol]/d for women and <2 drinks/d for men), and meeting at least 3 of the 6 dietary recommendations by the World Cancer Research Fund/American Institute for Cancer Research Third Expert Report 2018, which included red meat <0.5 serving/d, processed meat <0.2 serving/d, dietary fiber ≥30 g/d, dairy products ≥3 servings/d, whole grains ≥48 g/d or account for at least half of total grains, and calcium supplement use. Multivariable Cox proportional hazards regression was used to calculate the hazard ratios (HRs) and 95% confidence intervals (CIs) while adjusting for age, calendar period, sex, ethnicity, current multivitamin use, regular aspirin use, family history of colorectal cancer, menopausal status and hormone use (women only).

24. 5.      Was there a difference in risk of a colon cancer diagnosis between individuals receiving one or more negative screening colonoscopies over time?

We agree this is an interesting and important question, but is beyond the scope of the current study. There have been numerous studies assessing CRC risk after negative screening, several examples are:

- Brenner, H., Haug, U., Arndt, V., Stegmaier, C., Altenhofen, L., & Hoffmeister, M. (2010). Low risk of colorectal cancer and advanced adenomas more than 10 years after negative colonoscopy. Gastroenterology, 138(3), 870-876.
- Singh, H., Nugent, Z., Mahmud, S. M., Demers, A. A., & Bernstein, C. N. (2010). Predictors of colorectal cancer after negative colonoscopy: a population-based study. American Journal of Gastroenterology, 105(3), 663-673.
- Brenner, H., Chang-Claude, J., Seiler, C. M., & Hoffmeister, M. (2011). Long-term risk of colorectal cancer after negative colonoscopy. Journal of Clinical Oncology, 29(28), 3761-3767.
- Samadder, J. N., Pappas, L., Boucherr, K. M., Smith, K. R., Hanson, H., Fraser, A., ... & Curtin, K. (2017). Long-term colorectal cancer incidence after negative colonoscopy in the state of Utah: the effect of family history. American Journal of Gastroenterology, 112(9), 1439-1447.
- Lakoff, J., Paszat, L. F., Saskin, R., & Rabeneck, L. (2008). Risk of developing proximal versus distal colorectal cancer after a negative colonoscopy: a population-based study. Clinical Gastroenterology and Hepatology, 6(10), 1117-1121.
- Singh, H., Turner, D., Xue, L., Targownik, L. E., & Bernstein, C. N. (2006). Risk of developing colorectal cancer following a negative colonoscopy examination: evidence for a 10-year interval between colonoscopies. Jama, 295(20), 2366-2373.
- Lee, J. K., Jensen, C. D., Levin, T. R., Zauber, A. G., Schottinger, J. E., Quinn, V. P., ... & Doubeni, C. A. (2019). Long-term risk of colorectal cancer and related deaths after a colonoscopy with normal findings. JAMA internal medicine, 179(2), 153-160.

25. 6.      Page 8: Participants were considered as endoscopically unscreened until the first time they reported undertaking endoscopy for screening purpose and as endoscopically screened thereafter… How did authors account for diagnostic colonoscopies performed for complaints that did not provide any pathologies? A person undergoing colonoscopy for other reason than screening with no significant findings will usually not undergo screening colonoscopy within the next couple of years.

Please see our response to the Comment 21.

-----------------------------------------------------------
