# Supplementary material for: Healthy lifestyle, endoscopic screening, and colorectal cancer incidence and mortality in the United States: A nationwide cohort study
Source: PLoS Med. 2021 Feb 1;18(2):e1003522. doi: 10.1371/journal.pmed.1003522 (PMC7886195; doi:10.1371/journal.pmed.1003522)
Supplement: Attachment — Submitted filename: Response to editors and reviewers.docx [file pmed.1003522.s018.docx]

We appreciate the opportunity to submit this revised manuscript to *PLOS Medicine*. We have addressed the editors’ and reviewers’ comments as detailed below in our point-by-point response. We hope that the revised manuscript will be suitable for publication and we welcome any additional feedback.

-----------------------------------------------------------

Requests from the editors:

1. Please compare the spline fit and linear fit as suggested by Ref1 in order to check what the differences are and if they are of practical importance rather than statistical significance. Please look at the reduction in error between the two models and then judge whether it is big enough to make the more complex spline model worth the effort.

Thanks for the thoughtful comments. As shown in Figure 1 (copied below), we modeled the lifestyle score as both a continuous and categorical variable and presented the hazard ratios for both analyses. As per the reviewer #1’s prior comment, we used the spline model to test any potential non-linear relationship between the lifestyle score and colorectal cancer incidence and mortality. We agree that it should be the model fit/effect size rather than the P value that determines whether the spline model is worth the effort. To clarify this, we compared the goodness-to-fit statistic, -2 Log Likelihood (-2LL), between the linear and spline models. To enable this comparison, we forced the spline terms into the model and indeed observed a minimal improvement in the model fit, with the -2LL=25771.0 for the linear model and 25769.6 for the spline model in the incidence analysis and 8858.3 for the linear model and 8856.7 for the spline model in the mortality analysis. These results again support our notion that there did not seem to be a strong non-linear relationship between the lifestyle score and colorectal cancer incidence and mortality. Therefore, to avoid any unnecessary complexity and confusion, we have removed the spline analysis from this revised manuscript and based our findings on the main analysis in Figure 1.

2. Please add "our results indicate" or "our study suggests" in order to tone down the language throughout.

To address this, we have added "our results indicate" or "our study suggests" in appropriate locations throughout the text, including:

(Abstract, Page 3)

**Conclusions:** Our study suggests that healthy lifestyle may be associated with lower CRC incidence and mortality independent of endoscopic screening.

(Author Summary, What do these findings mean, Page 5)

Our results indicate that healthy lifestyle may be associated with lower colorectal cancer incidence and mortality independent of endoscopic screening status.

(Discussion, Page 15)

If these associations were causal, our results suggest that approximately 32% of CRC cases and 34% of CRC deaths could potentially be prevented by endoscopic screening alone and …

(Discussion, Page 16)

For example, compared to individuals with no endoscopic screening or healthy lifestyle, those with the five healthy lifestyle practices but remain unscreened might have a 62% reduction in CRC risk (HR, 0.38; 95% CI, 0.26-0.56), which was very close to that (63%) among individuals only undertaking endoscopic screening but still living an unhealthy lifestyle (HR, 0.37; 95% CI, 0.24-0.56).

(Discussion, Page 18)

In conclusion, our study suggests that adherence to healthy lifestyle is associated with substantial reduction in CRC incidence and mortality independent of endoscopic screening.

3. Please avoid implying a causal relationship between healthy lifestyle, endoscopic screening and colorectal cancer as this is an observational study

To address this, we have revised our statements throughout the text to avoid implying a causal relationship, including:

(Author Summary, What did the researchers do and find, Page 5)

We also found that an integration of healthy lifestyle with endoscopic screening might substantially enhance the prevention of colorectal cancer and related death compared with endoscopic screening alone, and the increment of the beneficial association was particularly high for proximal colon cancer.

(Author Summary, What do these findings mean, Page 5)

Our results indicate that healthy lifestyle may be associated with lower colorectal cancer incidence and mortality independent of endoscopic screening status.

(Results, Page 14)

For mortality, combining a healthy lifestyle (score ≥4) with endoscopic screening was associated with an increase in the PAR from 13% (95%, 2-23%) to 52% (95% CI, 20-74%) for proximal colon cancer, and from 49% (95% CI, 42-56%) to 65% (95% CI, 48-78%) for distal CRC.

(Results, Page 14)

In the stratified analysis, we found that an integration of healthy lifestyle with endoscopic screening was associated with a substantially increased PAR across all the subgroups …

(Discussion, Page 15)

To the best of our knowledge, the current study represents the first effort to answer that question and to comprehensively quantify the joint beneficial association of healthy lifestyle and endoscopic screening with CRC prevention.

(Discussion, Page 16)

Therefore, given the independent pathways through which healthy lifestyle and endoscopic screening exert protection against CRC, it is not surprising that we observed a similar CRC-preventive association of adherence to healthy lifestyle in individuals with and without endoscopic screening.

(Discussion, Page 16)

… the fact that healthy lifestyle may be associated with a further increased CRC prevention highlights the need to promote healthy lifestyle to optimize CRC prevention.

(Discussion, Page 16)

Second, we observed a higher increment in the beneficial association for proximal colon cancer when integrating healthy lifestyle with endoscopic screening.

4. Please introduce all acronyms on first view. For instance the study cohorts are mentioned in the methods but it is not clear what the "NHS" stands for, for example.

The acronym of NHS was first introduced in the last paragraph of Introduction, which is before the Methods section. But we have double checked the manuscript throughout to make sure all acronyms have been introduced on their first view.

5. Please add participant demographics in the abstract

Since the sex distribution of participants was already stated in the first sentence in the Abstract Methods and Findings, we have addressed this comment by adding a description of the participants’ age.

(Abstract, Page 2)

We calculated hazard ratios (HRs) and population-attributable risks (PARs) for CRC incidence and mortality in relation to healthy lifestyle score according to endoscopic screening. Participants’ mean age (standard deviation) at baseline was 54 (8) years. During a median of 26 years (2,827,088 person-years) follow-up, we documented 2,836 incident CRC cases and 1,013 CRC deaths.

Comments from the reviewers:

Reviewer #1: The authors have addressed most of my concerns. The remaining issue is about the splines.

I checked "proceed without recommendation" because what to do depends on non-statistical issues.

Here is the statistics part:

6. The authors tested a cubic spline for statistical significance and found it was not significant. But the p value of a test for linearity is not really the point. P values, in general, are problematic (see the American Statistical Ass'n Statement https://medium.com/@peterflom/some-thoughts-on-9-11-4f746ae9bdcb) and, here, as often, the real issue is effect size. That is, are the curves shown in the figure in their response to me straight or not?

The same effect size will be significant with more subjects. The key issue is whether it is meaningful and important.

And that's a non-statistical question. It depends on whether the difference between a straight line estimate and a spline estimate is "worth it" in terms of complexity.

One way to help answer this question is to see how well the two models do - how much does the spline model reduce the error? Is it enough that people in the field will care?

Thanks for the reviewer’s comments. We agree and have made revisions accordingly. The details have been described in our response to the Editor’s Comment.

7. Reviewer #3: The revised version of the manuscript has been strongly improved. My questions have been sufficiently answered.

Thank you.

-----------------------------------------------------------
