# Supplementary material for: Healthy lifestyle, endoscopic screening, and colorectal cancer incidence and mortality in the United States: A nationwide cohort study
Source: PLoS Med. 2021 Feb 1;18(2):e1003522. doi: 10.1371/journal.pmed.1003522 (PMC7886195; doi:10.1371/journal.pmed.1003522)
Supplement: Attachment — Submitted filename: Response to editors and reviewers.docx [file pmed.1003522.s019.docx]

We appreciate the opportunity to submit this revised manuscript to *PLOS Medicine*. We have addressed the editors’ comments as detailed below in our point-by-point response. We hope that the revised manuscript will be suitable for publication and we welcome any additional feedback.

------------------------------------------------------------
Requests from Editors:

1. Please revise to "United States" in the title

Revised.

2. Please capitalise "W" in whites

We have capitalized all the “W” in white or whites throughout the manuscript.

3. Page 6- paragraph 2 suggest revising “high quality diet” as it is currently ambiguous

We have revised the “high-quality diet” to be “healthy diet” throughout the manuscript.

4. Page 7 suggest removing “prospectively”

We have removed the “prospectively” in the last paragraph of Introduction on page 7.

5. Please add citations to or copies of the questionnaires used in both NHS and HPFS cohorts

The questionnaires used in the NHS and HPFS cohorts have been developed and updated over the years to accommodate changes in the exposure profiles and new research questions, thus we are afraid that there is no single citation for the questionnaires. But we have added a citation which summarizes the key information about the NHS cohort, and the methods and questionnaires are similar for the HPFS. We have always been willing to share our questionnaires and research tools with the research community.

(Methods, page 7)

In both cohorts, participants completed a detailed questionnaire about their lifestyle and medical information at baseline, and every two years thereafter, with over 90% of follow-up [18].

18.	Bao Y, Bertoia ML, Lenart EB, Stampfer MJ, Willett WC, Speizer FE, et al. Origin, Methods, and Evolution of the Three Nurses' Health Studies. Am J Public Health. 2016;106(9):1573-81. Epub 2016/07/28. doi: 10.2105/ajph.2016.303338. PubMed PMID: 27459450; PubMed Central PMCID: PMCPMC4981810.

6. Page 12 Results 1st paragraph should say “multivitamins”

We have revised all the “multivitamin” to be “multivitamins” throughout the manuscript, including 7 in the main manuscript and 14 in the Supporting Information.

7. Page 16- please remove "remarkable"

We have removed the “remarkable” in the fourth paragraph of Discussion on page 16.

8. Discussion-I suggest removing all iterations of “causal” as this is an observational study. In addition, I suggest removing the speculation about biological pathways linking healthy lifestyle and CRC incidence.

We have removed all “causal” throughout the manuscript, including on the pages of 4, 11, 15, 16, and 18.

To avoid the impression that the biological pathways lack evidence, we have removed the “putatively” in the sentence.

(Discussion, page 16)

On the other hand, lifestyle modification, as a primary prevention approach for CRC, ~~putatively~~ operates through various biological pathways, including reduction in hyperinsulinemia and systemic inflammation, and modulation of gene expression and the gut microbiota.

9. Late in the paper you note that colonoscopy can include removal of CRC precursors. Please mention this as a possible basis for the preventive effect in the author summary and/or introduction. I assume there are no data on this.

We have add this statement in the author summary and Introduction.

(Author Summary, page 4)

Endoscopic screening can detect and remove precancerous lesions, but it remains unknown whether individuals having undergone endoscopic screening can still benefit from healthy lifestyle.

(Introduction, page 6)

Screening has substantially contributed to the decline in CRC incidence and mortality in elderly Americans during the past two decades through detection and removal of precancerous lesions.

10. Please remove funding and conflicts of interest information from the main text. These should be provided in the relevant sections of the article submission form.

We have removed the fundings and conflicts of interest from the main text and provided them in the submission form.

11. Presumably, those less likely to be screened are less likely to have a healthy lifestyle - please mention this as a possible limitation?

We have added this as a potential limitation.

(Discussion, page 18)

Third, our study participants are all health professionals and predominantly Whites, thereby limiting the generalizability of our findings, particularly given that individuals with a healthier lifestyle are more likely to undertake screening.

12. Discussion- please avoid labels like “unhealthy lifestyle” I suggest replacing with less healthy lifestyle or similar

We have revised all the “unhealthy lifestyle” to be “less healthy lifestyle” throughout the manuscript, including on the pages of 13 and 17.

13. STROBE checklist- some information appears to be missing, for example page 3

We have refilled the STROBE checklist table.

Comments from Reviewers:

14. Reviewer #1: The authors have addressed my concerns and I now recommend publication

    Thank you.


    Any attachments provided with reviews can be seen via the following link:

------------------------------------------------------------
